# New Trends in Nucleoside Biotechnology

**Published:** 2010-07

**Authors:** I.A. Mikhailopulo, A.I. Miroshnikov

**Affiliations:** Institute of Bioorganic Chemistry, National Academy of Sciences, Belarus; Shemyakin and Ovchinnikov Institute of Bioorganic Chemistry, Russian Academy of Sciences

**Keywords:** nucleosides, nucleic acid metabolism enzymes, chemoenzymaticsynthesis, bio-mimetic synthesis

## Abstract

This review focuses on new trends in nucleoside biotechnology, which have emerged during the last decade. Continuously growing interest in the study of this class of compounds is fueled by a number of factors: ( * i * ) a growing need for large–scale production of natural 2 ′ –deoxy– β –D–ribonucleosides as well as their analogs with modifications in the carbohydrate and base fragments, which can then be used for the synthesis and study of oligonucleotides, including short–interfering RNA (siRNA), microRNA (miRNA), etc.; ( * ii * ) a necessity for the development of efficient practical technologies for the production of biologically important analogs of natural nucleosides, including a number of anticancer and antiviral drugs; ( * iii * ) a need for further study of known and novel enzymatic transformations and their use as tools for the efficient synthesis of new nucloside analogs and derivates with biomedical potential. This article will review all of these aspects and also include a brief retrospect of this field of research.

## INTRODUCTION

**Scheme 1:**
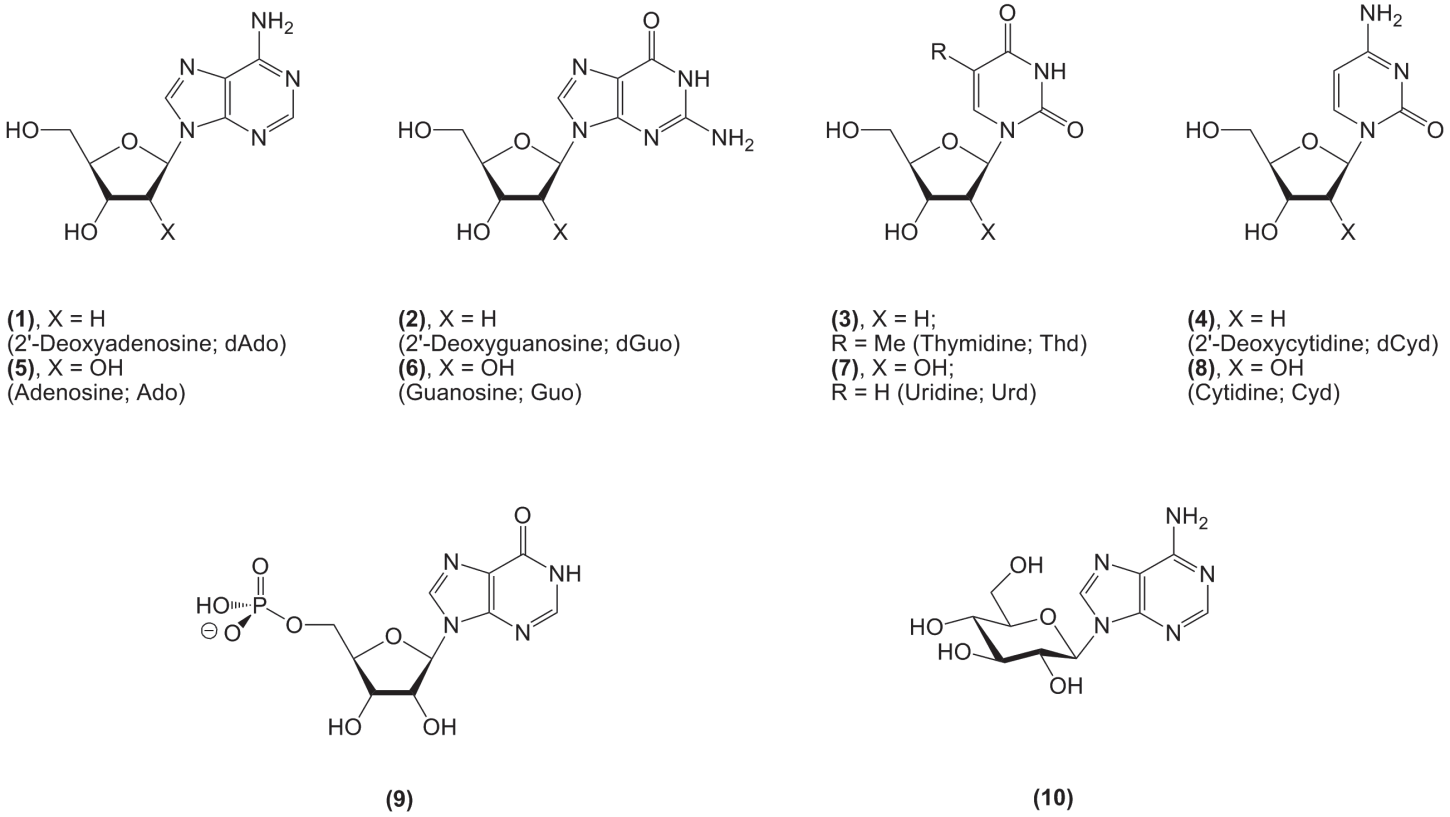


Nucleosides are a large family of natural compounds and their chemically modified analogs, which are characterized by great structural diversity. The four 2 ′ –deoxy– β –D–ribonucleosides of adenine ( ** 1 ** ) and guanine ( ** 2** ), and thymine ( ** 3 ** ) and cytosine ( ** 4 ** ), along with the related four β –D–ribonucleosides ( ** 5 ** – ** 8** ), are the main constituents of DNA and RNA, respectively (Scheme 1). Analogs of these natural nucleosides with variously modified carbohydrate and/or aglycon fragments have been found in RNA’s and are also included into a sub–family of nucleoside antibiotics which is also characterized by great structural diversity. 5’–Phosphorylated nucleosides, called nucleotides, are important metabolites of DNA and RNA biosynthesis, and they also act as co–substrates and cofactors of a large number of biochemical transformations.

The first identified natural representative of this family, inosine–5’–monophosphate ( ** 9 ** ; IMP; the name inosine originates from the Greek word * inos * – muscle), was isolated from beef extract by J.F. von Liebig in 1847. He also described its taste intensifier property; synthesis of IMP from inosine and its structure as ribofuranoside 5’–monophosphate was described by P.A. Levene & R.S. Tipson 88 years later (Scheme 1) [1]. It is interesting to note that it was P.A. Levene who coined the general term “nucleotide” for phosphoric acid derivatives formed as the result of nucleic acid hydrolysis, and suggested the term “nucleoside” for dephosphorylated nucleotides, and also identified * D * –ribose and later 2–deoxy– * D * –ribose as constituents of RNA and DNA, respectively [2 – 7].

Pioneering structural studies on nucleosides and nucleotides during the last decade of the 19th and first decade of the 20th centuries showed that DNA and RNA consist of five heterocyclic bases and two pentoses. The first chemical condensation of these two of two components was reported by E. Fischer & B. Helferich in 1914 [8]; condensation of a silver salt of 2,8–dichloroadenine with 2,3,4,5–tetra– * O * –acetyl– α – * D* –glucopyranosyl bromide followed by deprotection and hydrodehalogenation yielded a nonnatural nucleoside * N *^ 9 ^ –( β – *D * –glucopyranosyl)adenine ( ** 10 ** ), whose structure was unequivocally proved by J.M. Gulland & L.F. Story 24 years later (Scheme 1) [9]. Between World War I and II, a number of very important studies dedicated to the chemical synthesis of pyrimidine and purine nucleosides were published, but systematic studies on the chemical synthesis of nucleosides, nucleotides, and oligomers were started by A. Todd and his co–workers in 1942 at Cambridge University in England and somewhat later in the USA. Since then, numerous books and reviews have been published on the subject, summarizing the enormous progress achieved (see [10 – 12]).

Systematic studies of the biological properties of nucleosides began in the second half of the 1940s. Somewhat earlier, P. Fildes & D.D. Woods formulated the antimetabolite theory and a resulting approach to the design of natural compound analogs with biomedical potential sparked an enormous amount of research in this area (for the relevant reviews, see [13, 14]). Despite the moderate predictive power of this theory, synthesis of a large variety of natural nucleoside analogs and data on their biological properties yielded ( * i * ) very useful tools for studying biochemical transformations, which facilitated understanding of the mechanism of functioning of enzymes of nucleic acids metabolism; ( * ii * ) an analysis of the structure–activity relationships, which allowed rational design of new analogs with improved activity–toxicity ratios; and ( * iii * ) a number of anticancer and antiviral drugs.

**Scheme 2:**
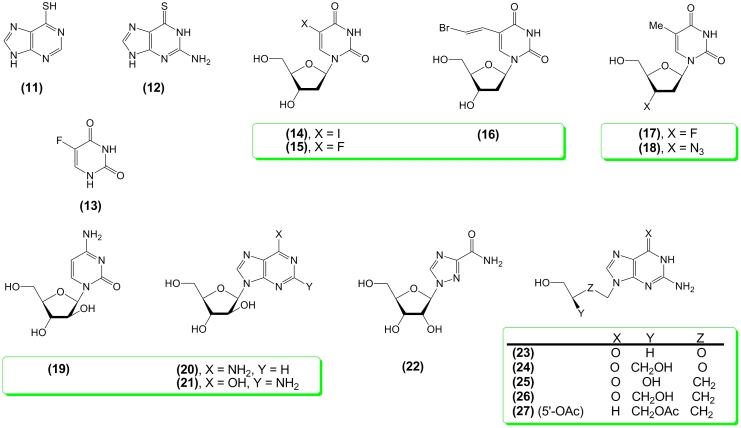


Thirty years of systematic studies resulted in the discovery of several major structures of great biological and medicinal importance, such as heterocyclic bases (6–mercaptopurine **(11) ** , thioguanine ** (12) ** , 5–fluorouracil ** (13)** ), analogues of thymidine modified at C5 of an aglycone (2 ′ –deoxy–5–iodouridine ** (14), ** Idoxuridine,; Iduviran;, 2 ′ –deoxy–5–fluorouridine ** (15), ** FUDR, Floxuridine; ( *E * )–5–(2–bromovinyl)–2 ′ –deoxyuridine **(16) ** , BVDU, Brivudine) and at C3 ′ of the carbohydrate moiety (3 ′ –deoxy–3 ′ –fluorothymidine ** (17), ** FLT, Alovudine and 3 ′ –deoxy–3 ′ –azidothymidine ** (18), ** AZT, Zidovudine), β –D–arabinofuranosyl nucleosides (1–( β – *D * –arabinofuranosyl)–cytosine ** (19), ** aC, Cytarabine; –adenine ** (20), ** aA, Vidarabine; –guanine ** (21),** aG), 3–carboxamido–1–( β – * D* –ribofuranosyl)–1,2,4–triazole ** (22), ** Virazole, Ribavirin, hyper–modified purine acyclonucleosides, and also analogs in which the sugar moiety of a nucleoside is replaced with an aliphatic chain mimicking the carbohydrate fragment, such as Aciclovir ** (23), ** ACV, Zovirax; Gancyclovir ** (24), ** DHPG, Cytovene; Buciclovir ** (25) ** , Penciclovir ** (26), ** and Famciclovir ** (27)** (Scheme 2).

Discovery of a number of compounds that displayed strong antiviral and/or anticancer activities, some of which were later approved by the FDA (Food and Drug Administration, USA), as well as isolation of nucleoside antibiotics from natural sources [15], stimulated extensive synthesis of a wide variety of modified nucleosides. Studies aimed at shedding light on the mechanisms behind the antiviral and antitumor activities of these compounds yielded extensive data regarding the metabolic transformations of modified nucleosides, including their ** metabolic activation and deactivation. Moreover, these studies identified the enzymatic reactions involved in these activities, and also led to the discovery of the role of nucleoside utilization mechanisms (“salvage” synthesis) and the involvement of virus–encoded nucleoside kinases in a key step of nucleoside activation via intracellular 5 ′ –monophosphorylation. The nucleoside–5’–monophosphates are then further metabolized into 5’–di– and 5’–triphosphates, which can then take part in various metabolic transformations [14, 16 – 19].

It was established that the majority of nucleoside analogs exhibiting antiviral and/or antitumor activities are not active as such but gain activity after being transformed into nucleotides by intracellular enzymes. In the case of antiviral agents, nucleoside–5’–triphosphates are often true inhibitors of viral DNA or RNA polymerases. In some cases, polymerases introduce an analog of the natural substrate into the growing chain, thus blocking or severely impeding the chain’s growth or producing a functionally incompetent biopolymer [16, 17].

In cancer cells, synthesis of the active species is initiated either by the transformation of a heterocyclic base into the respective ribonucleoside–5 ′ –monophosphate, catalyzed by nucleoside phosphoribosyl transferase or by direct 5 ′ –monophosphorylation of nucleosides by cellular nucleoside kinases [14, 20 – 22]. On the contrary, in virus–infected cells, the first critical step of antiviral nucleoside activation involves 5 ′ –monophosphorylation catalyzed by virus–encoded kinases [16, 17, 23].

Catabolic deactivation of biologically active nucleosides often involves deamination of cytosine and adenine nucleosides by the respective deaminases, which usually yield inactive derivatives [14, 24, 26], and phosphorolytic cleavage of the glycoside bond by nucleoside phosphorylases, which results in the formation of heterocyclic bases and α – * D * –pentofuranose 1–phosphates [24, 27 and works cited in 27].

**Scheme 3:**
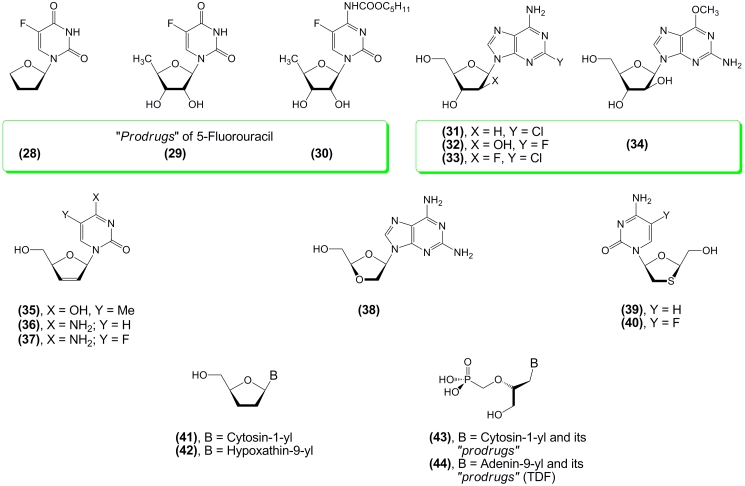


New data on the metabolism of nucleosides and their mechanism of action towards their targets allowed improvement of the activity of the originally discovered compounds by protecting them from catabolic transformations and facilitating their targeted delivery, and also stimulated the search for new biologically active molecules [18, 19, 28]. The first approach can be illustrated by the anticancer drugs Ftorafur^®^ **(28)** , 5–fluoro–5’–deoxyuridine ** (29), ** and Capecitabine ** (30) ** ; by nucleosides similar to aA, such as Cladribine ** (31)** , Fludarabine ** (32) ** and Clofarabine ** (33) ** , which are highly active against various forms of leukemia and are resistant to deamination by adenosine deaminase [21, 22]; and by the antileukemic drug Nelarabine ** (34),** “prodrug” aG, which has better solubility and stronger activity as compared to the parent aG drug [29] (Scheme 3).

Elucidation of the mechanism of AZT action and establishment of viral–encoded reverse transcriptase as an important biochemical target for anti–HIV drugs stimulated extensive synthesis of various 2 ′ ,3 ′ –dideoxy nucleosides, * e.g * ., Zalcitabine ( ** (41) ** ; Hivid®), Didanosine ** (42) ** and related nucleosides with a C2 ′ –C3 ′ double bond, 2’,3’–didehydro–2’,3’–dideoxythimidine ( ** (35)** , Stavudine, Zerit®) and its cytosine analogs ( ** (36), (37) ** ; Reverset™), nucleosides with oxygen or sulfur atoms substituting the C3’ carbon atom of the pentofuranose ring ( ** (38), ** Amdoxovir; ** (39),** Lamivudine, Epivir®; ** (40) ** , Emtricitabine, Emtriva®), as well as a number of hypermodified acyclic nucleosides with phosphonate function and their prodrugs ( ** (43),** Cidofovir; ** (44), ** Tenofovir) [30 – 34] (Scheme 3).

Notably, pioneering studies by H. Schaeffer ** and co–workers on the synthesis and study of the biochemical properties of acyclic nucleosides led to the discovery of 9–[2–hydroxy(ethoxymethyl)]guanine ( ** (23) ** , Acyclovir) as an effective antiviral drug [35 – 38] and stimulated extensive synthesis of a wide variety of acyclic nucleosides modified either in the aglycone or in the acyclic fragment t, including phosphonate analogs of nucleoside 5 ′ –phosphates and their numerous prodrugs, some of which manifested a broad spectrum of biological activities [39].

Up to the present, a vast majority of the modified nucleosides have been synthesized by chemical methods. Most of the developed synthetic approaches can be divided into three main groups: ( * i * ) convergent synthesis, employing the suitable sugar or sugar–mimicking derivatives as glycosylating agents, ( * ii * ) chemical transformations of natural nucleosides, and ( * iii * ) rational combinations of both aforementioned approaches. Despite the impressive progress achieved in the development of chemical methods, production of many antiviral and anticancer drugs, as well as other biologically active compounds, remains a challenge. This leads to high drug costs and consequently prevents extensive biological trials and studies, as well as broad therapeutic application. The need for the development of new strategies became apparent in the late 1970s.

## Chemo – enzymatic strategy for the synthesis of nucleosides (nucleoside biotechnology)

Amidst the great number of nucleic acid metabolism enzymes, approximately 20 are promising in relation to the development of novel effective strategies for the production of biologically important nucleosides. These are foremostly enzymes that catalyze the condensation of heterocyclic bases and sugars, thus forming glycoside bonds, and also enzymes that are involved in various transformations of nucleosides. These enzymes are of utmost importance for the research and development of novel approaches for nucleoside synthesis.

In parallel with the pioneering chemical studies and investigation of the biochemical properties of modified nucleosides, researchers began attempting the isolation of enzymes involved in nucleic acid metabolism from natural sources and to study the mechanisms of their functioning (for a review, see [24]). The first reports by P. Levene ** and co–workers [40 – 44] and W. Jones [45, 46] on the activities involved in nucleic acid hydrolysis and nucleoside disassembly were published in 1911; later on, Levene and co–workers described a procedure for the isolation of “nucleosidase” from the spleen, kidney, and pancreas of cattle. The isolated enzyme was able to hydrolyze adenosine and inosine in phosphate buffer with similar efficiency, thus yielding the respective bases and ribose (formation of ribose–1–phosphate was not discovered at that time!). The authors also investigated the properties of this enzyme [47 – 49]. They determined the optimal temperature (37 °C) and pH (7.5) of the reaction and found that ( * i* ) ribose and adenine exert “an impeding influence on the progress of the reaction,” ( * ii * ) kaolin completely adsorbs the partially purified enzyme from the solution, and the enzyme–kaolin complex is stable within pH 4.0 – 8.0 values at 40 °C for 15 h and shows the same level of activity, ( * iii * ) a chemically prepared adenine nucleoside containing hexose (the structure was not established) could not act as a substrate for this enzyme.

**Scheme 4:**
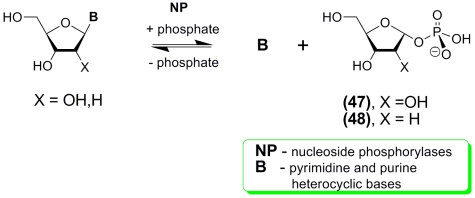


Later on, H. Kalckar investigated nucleosidase extracted from rat liver and found that ( *i * ) this enzyme was in fact a purine nucleoside phosphorylase **** (PNP; EC 2.4.2.1), which hydrolyzed inosine or guanosine via phosphorolysis, thus yielding ribose–1–phosphate (structure was later determined to be α – * D* –ribofuranose–1– * O * –phosphate; RP; **(47) ** ; for reviews, see [24, 50]) and the corresponding purine bases, hypoxanthine or guanine; ( * ii * ) when RP is incubated with hypoxanthine or guanine in the presence of PNP, a rapid formation of inosine or guanosine takes place [51, 52]. Kalkar’s paper was the first report on the isolation of pure RP. Shortly after this publication, L. Manson & J. Lampen showed that quiescent * Esherichia coli * cells, as well as a cell–free extract, contain enzymes which can hydrolyze 2 ′ –deoxyinosine and thymidine in the presence of inorganic phosphate down to free bases and a deoxyribose ester whose structure was later established to be 2–deoxy– α – * D* –ribofuranose–1–phosphate ** (48) ** [24, 53]. It was suggested that this extract contains purine and pyrimidine nucleoside phosphorylases, whose specificity was later found to be similar to that of mammalian enzymes. Moreover, evidence was presented that these bacterial enzymes reversibly catalyze the synthesis of nucleosides and their phosphorolytic degradation (Scheme 4).

Subsequent studies have corroborated and extended these fundamental findings, and it was found that purine nucleoside phosphorylase is specific to 9–( β –D–pentofuranosyl)purines, whereas mammalian PNP is specific to 6–oxopurines (compared with data by Levene * et al * . [47 – 49]; * vide supra* ) and their nucleosides, as well as some analogs, whereas PNP from bacterial sources displays very broad specificity, accepting both 6–oxo– and 6–aminopurines and their nucleosides as substrates, along with many analogs.

Thymidine phosphorylase (TP; EC 2.4.2.4) reversibly catalyzes the phosphorolysis of thymidine **(7) ** and 2 ′ –deoxyuridine but not uridine ** (7) ** or 1–( β – * D * –arabinofuranosyl)–thymine and –uracil, whereas uridine phosphorylase (UP; EC 2.4.2.3) does not distinguish between β – * D * –ribofuranose and 2 ′ –deoxy– β – * D * –ribofuranose in pyrimidine nucleosides and accepts 1–( β – * D * –arabinofuranosyl)–pyrimidines as substrates as well. Cytosine and its nucleosides are not substrates either for TP or UP; however, we must note two peculiar observations. Firstly, PNP exhibited cytidine phosphorylase activity in some experiments [54]. Secondly, it was shown that human deoxycytidine kinase is a cytosolic enzyme that plays a key role in the activation of therapeutically relevant nucleoside analogs via their 5’–monophosphorylation, accomplishes phosphorolytic cleavage of 2’–deoxynuclosides, including 2 ′ –deoxycytidine into free heterocyclic bases and 2–deoxy– α – * D* –ribofuranose–1–phosphate [55].

The results of pioneering research unequivocally indicated a possibility for the enzymatic synthesis of nucleotides using purine or pirimidine heterobases as a starting point and α – * D * –pentofuranose–1–phosphate or another nucleoside as a carbohydrate fragment donor (see [24, 56] for a review). The first attempts to use enzymes for the synthesis of pyrimidine nuclesides were made by ** M. Friedkin & D. Roberts, who attempted to synthesize thymidine and related nucleosides [57, 58], and by R. Duschinsky & C. Heidelberger for the synthesis of 5–fluoro–2 ′ –deoxyuridine (FUDR, ( **15) ** ) and 2 ′ –deoxy– β – * D* –ribofuranosil–5–trifluoromethyl–urcail (CF_3_–dUrd) [59–64]. Interestingly, the first report of FUDR synthesis via enzymatic transfer of the 2–deoxyribofurnaose residues of thymidine onto 5–fluoro–uracil ** (13)** was published in **** 1957 [59]. A preparative–scale enzymatic process was later patented [60]. The same group of researchers described a chemical method for the synthesis of 5–fluoro–2’–deoxyuridine ( ** 15 ** ), and a low–yield enzymatic process for the synthesis of another anti–cancer nucleoside – CF_3_–dUrd using a cell–free extract of * E. coli* as a source of thymidine phosphorylase [36] (See [24, 56, 65–68] for reviews). Later on, a number of 5–modified uracil nucleosides, including 2 ′ –deoxy–5–iodouridine (( ** 14) ** ; 55%), 5–fluoro–2 ′ –deoxyuridine (( ** 15) ** ; 65%), and * Е* –5–(2–bromovinyl)–2 ′ –deoxyuridine (( ** 16) ** ; 61%), were obtained by using thymidine ( ** 3 ** ) or 2 ′ –deoxyguanisone ( ** 2 ** ) as donors of 2–deoxyribofuranose, the appropriate heterobases as acceptors, and selected BM–11 * E. coli * as a biocatalyst [69].

**Scheme 5:**
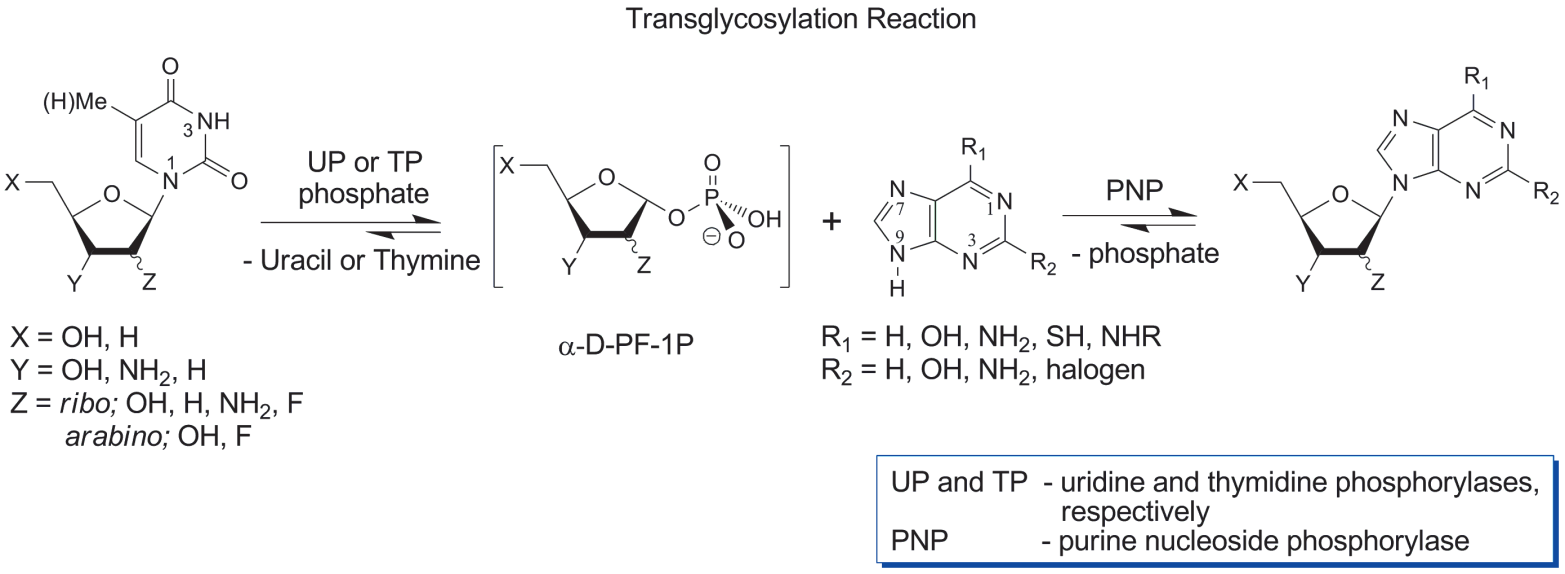


Transfer of a pentofuranose moiety from pyrimidine nucleosides to purine bases and *vice versa * (transglycosylation reaction) catalyzed by bacterial nucleoside phosphorylases (NP) was shown to be a very efficient method for the synthesis of a number of analogs of natural purine and pyrimidine nucleosides of biological and pharmaceutical importance. The most exploited pathway includes the transfer of a pentofuranase moiety from pyrimidine nucleosides to purine bases (Scheme 5).

This transglycosylation approach is based on numerous efficient chemical transformations of readily available natural pyrimidine nucleosides into diverse nucleosides modified in their carbohydrate component through the intermediate formation of * O *^ 2^ ,2 ′ (3 ′ ;5 ′ )– * anhydro * derivatives, followed by opening of the * anhydro * ring upon treatment with nucleophilic agents. Unfortunately, a similar approach is not practical for the production of related purine nucleosides. Moreover, distinctions in the substrate specificity of TP and UP extend the number of the pentofuranose donors of the transglycosylation reaction that can be used with the optimal efficiency. Thus, 1–(2–deoxy–2–fluoro– β – *D * –ribofuranosyl)uracil (Scheme 5, X = Y = OH; Z = * ribo* F) and 1–(2–deoxy–2–fluoro– β – * D* –arabinofuranosyl)thymine (Scheme 5, X = Y = OH; Z = * arabino* F) display no substrate activity towards UP: thus it cannot be employed as a biocatalyst; on the contrary, both nucleosides have been found to be (although very poor) substrates for TP, allowing their use for TP–catalyzed transglycosylation of purine bases [70, 71].

The successful employment of nucleoside phosphorylases as biocatalysts for the synthesis of purine arabinosides and a multitude of base– and carbohydrate–modified nucleosides has been described in numerous publications (for reviews, see [24, 56]).

Three types of biocatalysts have been successfully employed for transglycosylation reactions: ( * i * ) selected intact bacterial cells, which display UP and/or TP and PNP activities, ( * ii * ) intact bacterial cells overexpressing recombinant nucleoside phosphorylases, and ( * iii * ) purified recombinant enzymes.

Intact bacterial cells as a biocatalyst represent a kind of naturally immobilized enzyme, which can be used for the transformation of interest. Use of this type of biocatalysts offers some advantages (relatively low cost) over the application of purified enzymes or immobilized (encapsulated) enzymes. However, intact bacterial cells may display activities which will catalyze the transformation of the substrate and/or the desired product of the transglycosylation reaction into an undesirable form (see further). On the other hand, considerable progress has been achieved in the practical production of recombinant enzymes during the last decade, which makes these biocatalysts available for broad application, including the development of biotechnological processes for the production of drugs. In case of very low substrate activity of a pentofuranose donor or an acceptor base, use of purified enzymes as biocatalysts may be a rational alternative to the use of intact bacterial cells.

**Scheme 6:**
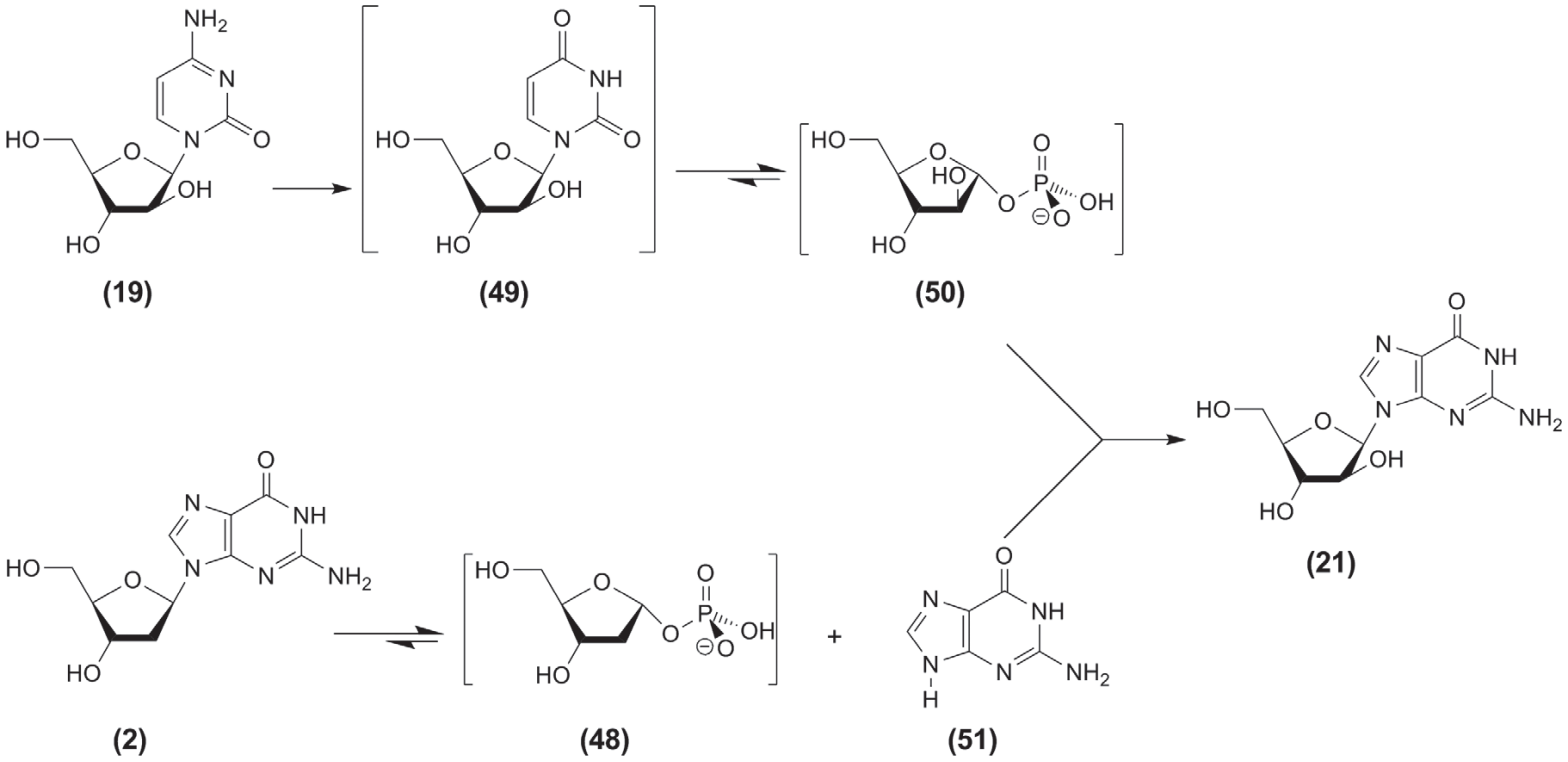


Notably, the off–pathway activities displayed by intact cells can be rationally involved in the synthesis of the desired nucleosides. For example, selected * E. coli* BM–11 cells displaying high cytidine deaminase (CDase) activity, along with UP and PNP activities, were employed as a biocatalyst for the synthesis of aG ** (21)** (isolated yield 48–53%) using aC ** (19) ** and 2 ′ –deoxyguanosine ( ** (2); ** dGuo) as donors of * D * –arabinofuranose residue and * in situ * formed guanine ( ** (51) ** ; Gua), respectively (Scheme 6). Deamination of aC to aU ** (49) ** by cytidine deaminase precedes the formation of α – * D * –arabinofuranose–1–phosphate **(50) ** from aU catalyzed by UP.

A similar approach was employed for the synthesis of 2 ′ –deoxy–2 ′ –fluoroguanosine using 2 ′ –deoxy–2 ′ –fluorocytidine as a donor of 2–deoxy–2–fluoro– α – * D* –ribofuranose–1–phosphate [73]; note that the use of selected * E. coli * BMT–4D/1A cells for the synthesis of 2 ′ –deoxy–2 ′ –fluoroguanosine [73] appears to be preferable over the use of purified UP and PNP [70, 71].

**Scheme 7:**
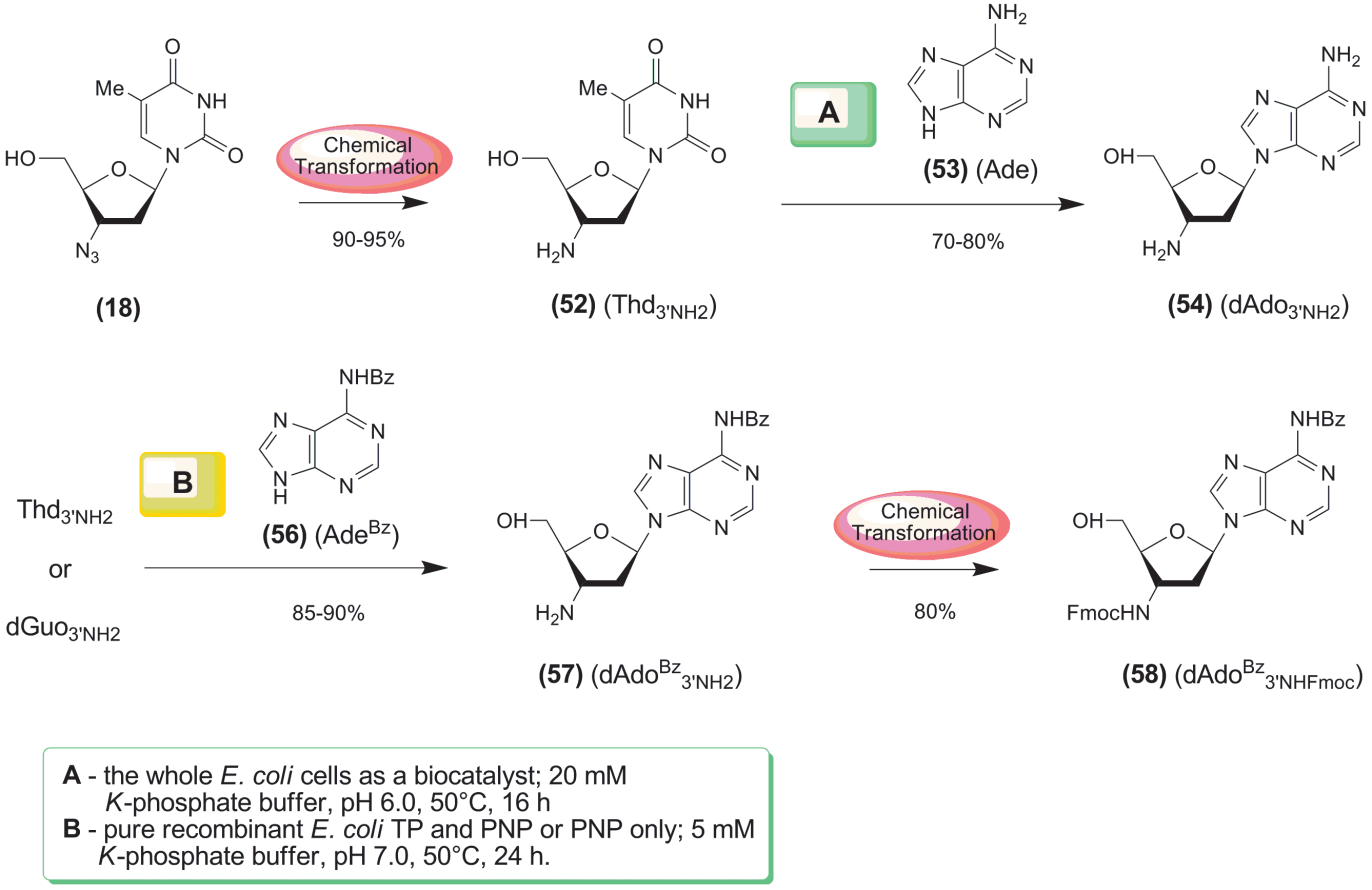


The use of the selected * E. coli * cells was found to be very efficient for chemoenzymatic syntheses of purine 3 ′ –amino–2 ′ ,3 ′ –dideoxy– β – * D * –ribonucleosides (Scheme 7) [74]. Notably, AZT ** (18) ** is neither a substrate for TP or UP and cannot, therefore, be used as a donor of the pentofuranose moiety. Reduction of the azido group of AZT produces 3 ′ –amino–2 ′ ,3 ′ –dideoxythymidine ( ** (52) ** ; dThd_3’NH2_) that is a satisfactory substrate for TP and can be used as a donor of the carbohydrate moiety. Transfer of the pentofuranose moiety from dThd_3’NH2_ to adenine catalyzed by intact *E. coli * cells proceeds smoothly, and the desired 3 ′ –amino–2 ′ ,3 ′ –dideoxyadenosine ( ** (54) ** ; ddAdo_3’NH2_) can be isolated with good yields. However, replacement of adenine **(53) ** by * N *^ 6 ^ –benzoyladenine ** (56)** in the aforementioned reaction produces 3 ′ –amino–2 ′ ,3 ′ –dideoxyadenosine ** (54) ** instead of the expected * N* ^6 ^ –benzoyl derivative of ddAdo_3’NH2_ **(57),** owing to the off–pathway activity present in the intact cells.

**Scheme 8:**
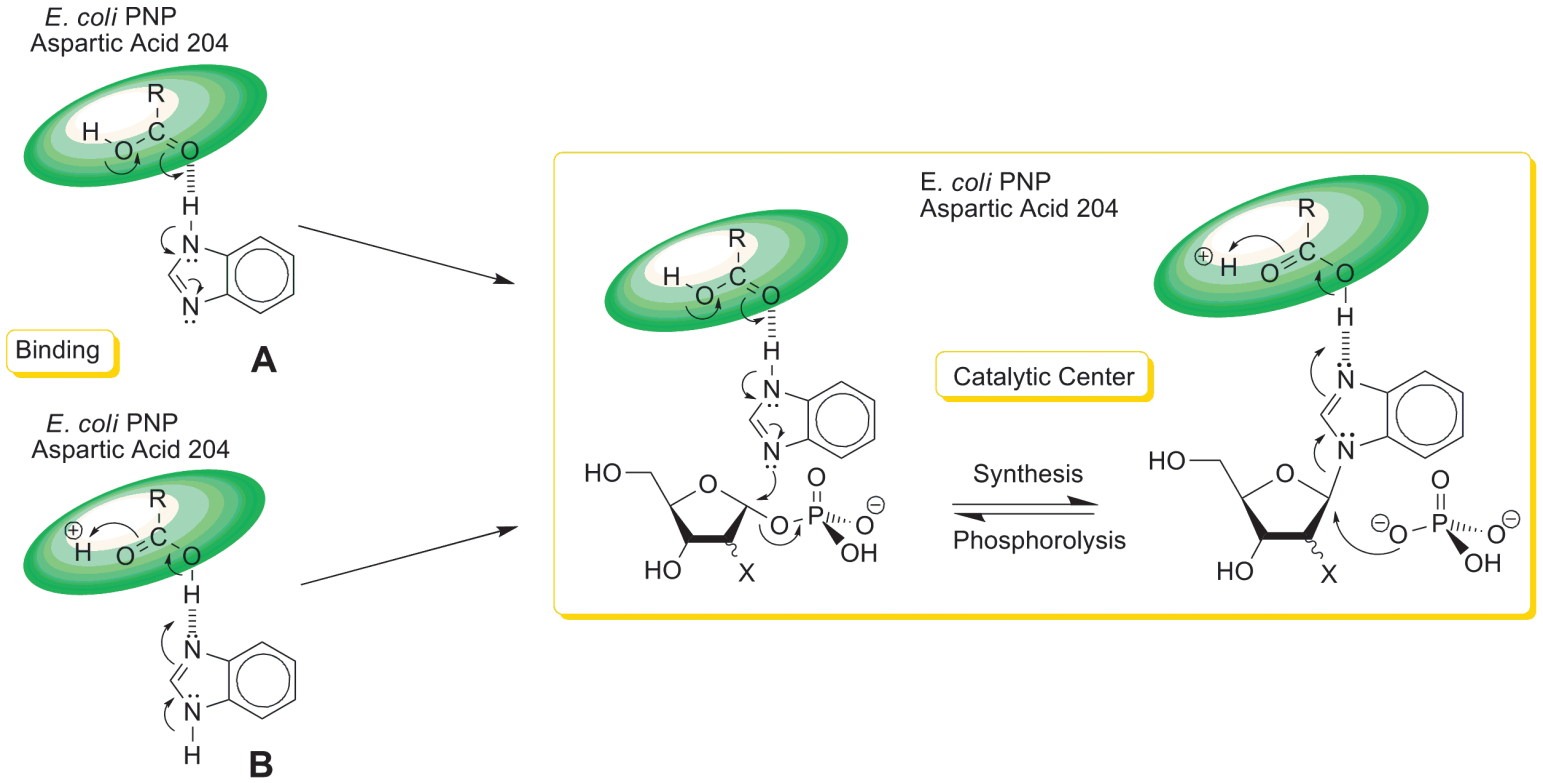


Taking into account that ddAdo_3’NH2_ with orthogonally protected amino functions ** (58) ** is of interest for oligonucleotide synthesis, we recently investigated transglycosylation reactions using pure recombinant * E. coli * TP and PNP [75]. It was found that the use of Thd_3’NH2_ as a donor of the pentofuranose residue and TP and PNP as biocatalysts or a dGuo_3’NH2_ / PNP combination (5 m М K–phosphate buffer ( рН 7.0), 50 ° С , 2 4 h ) produced the desired * N* ^6 ^ –benzoyl derivative of ddAdo_ 3’NH2_ **(57)** in high yield (Scheme 7) [76]. Standard treatment of the latter with Fmoc–OSU yielded the desired ddAdo^Bz^_3’NHFmoc_ **(58) ** with orthogonally protected amino groups.

The possible areas of application of nucleoside phosphorylases for the synthesis of nucleosides, as well as the limitations of this methodology, have been investigated in detail; however, several very interesting enzymatic synthetic reactions deserve special attention, because they are crucial for understanding the mechanism of synthetic reactions catalyzed by these enzymes and may expand the scope of their practical use.

It is well documented that the * N *^ 7 ^ –atom of purine plays a very important role in the phosphorolytic cleavage of the glycosyl bond of purine nucleosides ([77, 78] and works cited in [77]) and, it seems, in the reversed synthetic reaction catalyzed by * E. coli * PNP as well, even though the mechanism of this reaction has not been adequately studied. The finding that 3–deazapurines [79 – 81] and 1–deaza–, 3–deaza– and 1,3–dideazapurines (benzimidazoles, including fluoro–, chloro– and bromo–substituted) [82 – 84] are good substrates for * E. coli * PNP allows the authors to suggest a key role for two nitrogen atoms of the imidazole ring in the above–mentioned reaction. Namely, one of them is involved in the binding of the heterocyclic base in the enzyme’s active site, which may in turn increase the nucleophilicity of the second nitrogen atom. This facilitates an attack by this atom on the electrophilic * C *^ 1 ^ carbon atom of α – * D* –pentofuranose–1–phosphate and eventually results in the formation of a glycosidic bond (Scheme 8).

Remarkably, the mechanism of this synthetic reaction catalyzed by nucleoside phosphorylases did not attract the attention of researchers and many important details were left unclear. Thus, the mode of initial binding of the substrate or inhibitor of * E. coli* PNP (see binding types ** A ** and ** B ** in Scheme 8) might have shed light on the mechanism of the enzyme’s functioning and provided a clue for the explanation of some unusual observations. Participation of two nitrogen atoms in this reaction seems obvious taking into account the fact that 7–deazahypoxanthine ( ** (61) ** ; 7–DAH) is a very potent inhibitor of PNP (Scheme 8) [50, 85]. Tubercidine ** (59)** and 7–deazainosine ** (60) ** are not substrates for PNP and showed very low affinity for the active site of the enzyme. On the contrary, the free base, 7–deazahypoxanthine ** (61) ** , is recognized by the enzyme and forms a very strong PNP–phosphate–base complex, which results in complete inhibition of the enzyme [85].

**Scheme 9:**
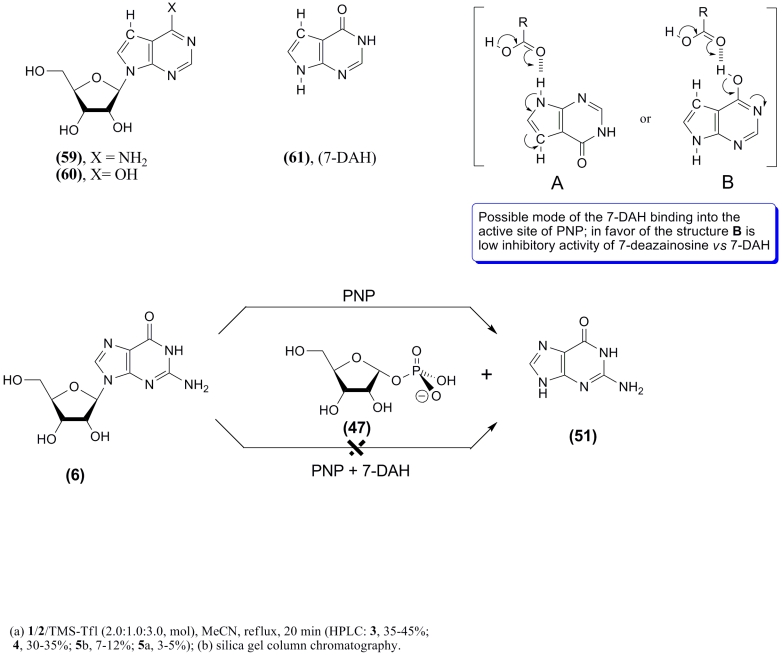


**Scheme 10:**
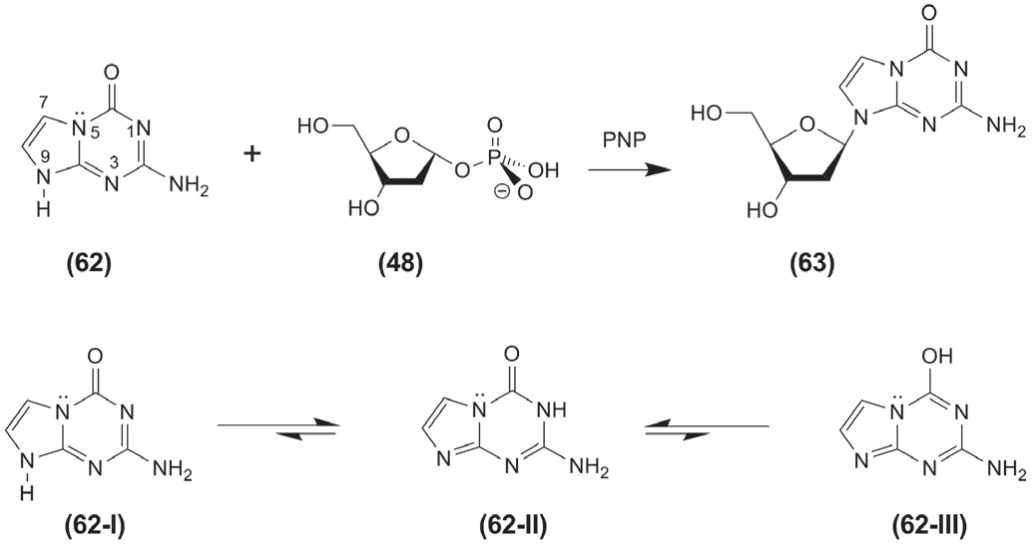


The mechanism of 7–DAH binding in the * E. coli * PNP binding site still remains unknown; two types of interactions can be proposed – ** А ** and ** В ** . The first (type ** A ** ) is similar to one of the two possible modes of binding of the natural substrate in the PNP active site via a hydrogen bond with the carboxyl moiety of aspartic acid–204 (type ** А ** on Scheme 8 **** and **** type ** A ** on Scheme 9). Obviously, type ** A ** binding of 7–DAH in the * E. coli* PNP binding site cannot result in the formation of a nucleoside, since there is no *N *^ 9 ^ –nitrogen atom (purine numbering). Type ** В ** binding involves the formation of an unusual hydrogen bond between the **** ОН –group of the tautomeric form of the cyclic amide. This hypothetic bond can seemingly stabilize the PNP–phosphate–7–DAH complex ( *N *^ 9 ^ –H–structure), whose electron or spatial structure either impedes or prevents a nucleophilic attack of the C 1–atom of α – * D * –pentofuranose–1–phosphate ( ** 47 ** ) (Scheme 9). The hypothetical possibility of the existence of a ** В** **– ** type structure is unexpectedly supported by the moderate acceptor activity of **** 5–aza–7–deazaguanine ( ** 62 ** ) during a glycosylation reaction involving PNP (bovine spleen extract; Sigma) and 2–deoxy– β – * D * –pentofuranose–1–phosphate ( ** 48** ) as a carbohydrate donor (see [86] and other works cited in this article). Indeed, the heterobase can exist in three tautomeric forms ( **62–I–III ** ), and one of them, a ( ** 62 ** – **III) ** structure, can be recognized by PNP and thus result in the formation of a nucleoside via a nucleophilic attack of the free * N *^ 9^ –nitrogen atom on the C 1–carbon atom of the carbohydrate substrate (Scheme 10). Notably, analysis of tautomeric structures involving * ab inito* (6–31G**) and semi–empirical methods ** (PM3, in water) (HyperChem 8.1) show that structure ** II ** is the most stable in terms of thermodynamics, while structures ** III ** and ** I ** are less stable (I.A. Mikhailopulo, unpublished).

Obviously, the binding mechanism of 7–DAH and 5–aza–7–deazaguanine ( **62 ** ) in the PNP active site and also the possible ways of using this information for the production of some 7–deazopurine–derived nucleosides deserve further thorough research.

**Scheme 11:**
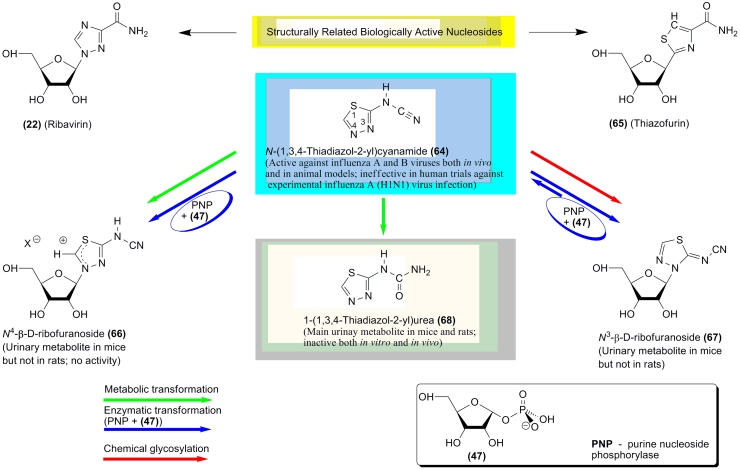


Other examples of unusual biotransformations are the metabolic and enzymatic transformations of the anti–influenza agent * N* –(1,3,4–thiadiazol–2–ylc)yanamide ( ** (64) ** ; LY217896) (Scheme 11) [87, 88]. This compound shows a degree of structural similarity with the heterocyclic bases of the antiviral nucleoside Virazole ( ** (22) ** ; Ribavirin) and of the anticancer C–nucleoside tiazofurin ** (65). ** It was found to be active against the influenza A and B viruses both * in vitro * and in animal models but was ineffective in clinical trials against an experimental influenza A (H1N1) virus. A number of its metabolites were detected in experiments with mammalian cells and animals as well, and the structures of three metabolites were established (Scheme 11).

It was found that purine nucleoside phosphorylases isolated from calf spleens and human erythrocytes, as well as the bacterial enzyme (Sigma, N–8265), catalyze the transformation of * N* –(1,3,4–thiadiazol–2–yl)cyanamide in the presence of α – *D * –ribofuranose–1–phosphate ** (47) ** into *N *^ 4 ^ – and * N *^ 3^ –ribosides (37 °C, 20–70 h, 2 – 200 units of PNP; the ratio between the *N *^ 4 ^ – and * N *^ 3^ –ribosides ( ** (66) ** and ** (67)) ** was found to be * ~* 1:3 (60–65% combined yield) at high concentrations of PNP and * ~* 3:1 (12–14% combined yield) at low concentrations of PNP) [89]. Interestingly, the formation of the mesoionic [88] or ionic (as shown in Scheme 11) * N* ^4 ^ –riboside ** (66) ** apparently proceeded in an irreversible manner, whereas the * N *^ 3 ^ –riboside ** (67) ** was found to be a substrate of PNP.

We must also mention several extremely interesting observations made in this excellent study. Firstly, 1,3,4–thiadiazol–2–ylcyanamide displayed broad antiviral activity *in vitro * and in animal models against orthomyxo– and paramyxoviruses. Oral, intraperitoneal or aerosol administration of the drug protected mice against lethal influenza A or B virus infections; however, it did not show either toxicity or anti–influenza activity in phase I trials on healthy volunteers [89]. Secondly, the data on the pharmacokinetics of the thiadiazol base also show considerable diversity. Thirdly, contrary to the above, PNP of mammalian and bacterial origin manifested close catalytic similarity in the ribosylation of this base, despite the well–known differences between the substrate preferences of these two types of PNP for natural substrates. These data imply that * N* –(1,3,4–thiadiazol–2–yl)cyanamide ** (64) ** , which does not have any common features with natural substrates of PNP, still possesses functionality that is sufficient for the synthetic reaction catalyzed by both types of PNP. Testing new heterocyclic bases as substrates of PNP may help understand this functionality, providing further insight into the mechanism of the enzyme’s function and also opening new possibilities for its practical use.

The use of intact bacterial cells as a biocatalyst for transglycosylation reactions (Scheme 5) implies that the cells contain uridine, thymidine, and purine nucleoside phosphorylases. Besides the aforementioned nucleoside phosphorylases, other phosphorylases have been found in bacteria that may be useful for the enzymatic synthesis of nucleosides. For instance, the nucleoside phosphorylase purified from the * Klebsiella * sp. strain LF1202 demonstrated very interesting properties [90]. It consists of five identical subunits with a molecular weight of 25 000 Da (based on the results of SDS–PAGE) and shows pyrimidine and purine nucleoside phosphorylase activities. Inosine, adenosine ** (5) ** , 2 ′ –deoxyadenosine ** (1) ** , guanosine ** (6), ** and 2 ′ –deoxyguanosine ** (2) ** showed similar substrate activity (relative activity * ~ * 100%) in phosphorolysis ( *K *_ m _ values for inosine and inorganic phosphate (P_i_) were calculated to be 0.66 and 0.56 mM, respectively); substrate activity for 2 ′ –deoxyinosine was 2.5–fold higher (254%); xanthosine and its 2 ′ –deoxy counterpart did not act as substrates. In the synthetic reaction, the substrate activity of hypoxanthine and adenine was similar ( * K *_ m _ values for hypoxanthine and α –D–ribofuranose–1–phosphate ( ** (47)** ; α – * D * –RF–1P) were calculated to be 0.45 and 0.14 microM, respectively); guanine showed somewhat decreased substrate activity in the synthetic reaction. As for pyrimidine nucleosides, uridine was found to be the best substrate (relative activity 368%) as opposed to 2 ′ –deoxyuridine (95%) and thymidine (29%); the * K *_ m _ values for uridine (0.38 mM) during phosphorolytic cleavage and for uracil (0.44 mM) during the synthetic reaction were similar. The substrate activity of uracil in the synthetic reaction with α – * D* –RF–1P (82%) and 2–deoxy– α – * D* –ribofuranose–1–phosphate ( ** 48 ** ) was found to be 82 and 39%, respectively; thymine showed decreased activity (17%); neither cytidine, nor 2 ′ –deoxycytidine, nor cytosine demonstrated any substrate activity in the enzymatic reactions.

The * Klebsiella * sp. nucleoside phosphorylase was employed for the synthesis of aA from aU and adenine (3:1 molar ratio) under optimized reaction conditions (0.1 M *K * –phosphate buffer, pH = 8.0; 6.7 mM concentration of adenine; 50 °C, 30 h; 0.86 units of enzyme) and converted approximately 90% of the adenine into aA, as assayed by TLC analysis of the reaction mixture [90].

**Scheme 12:**
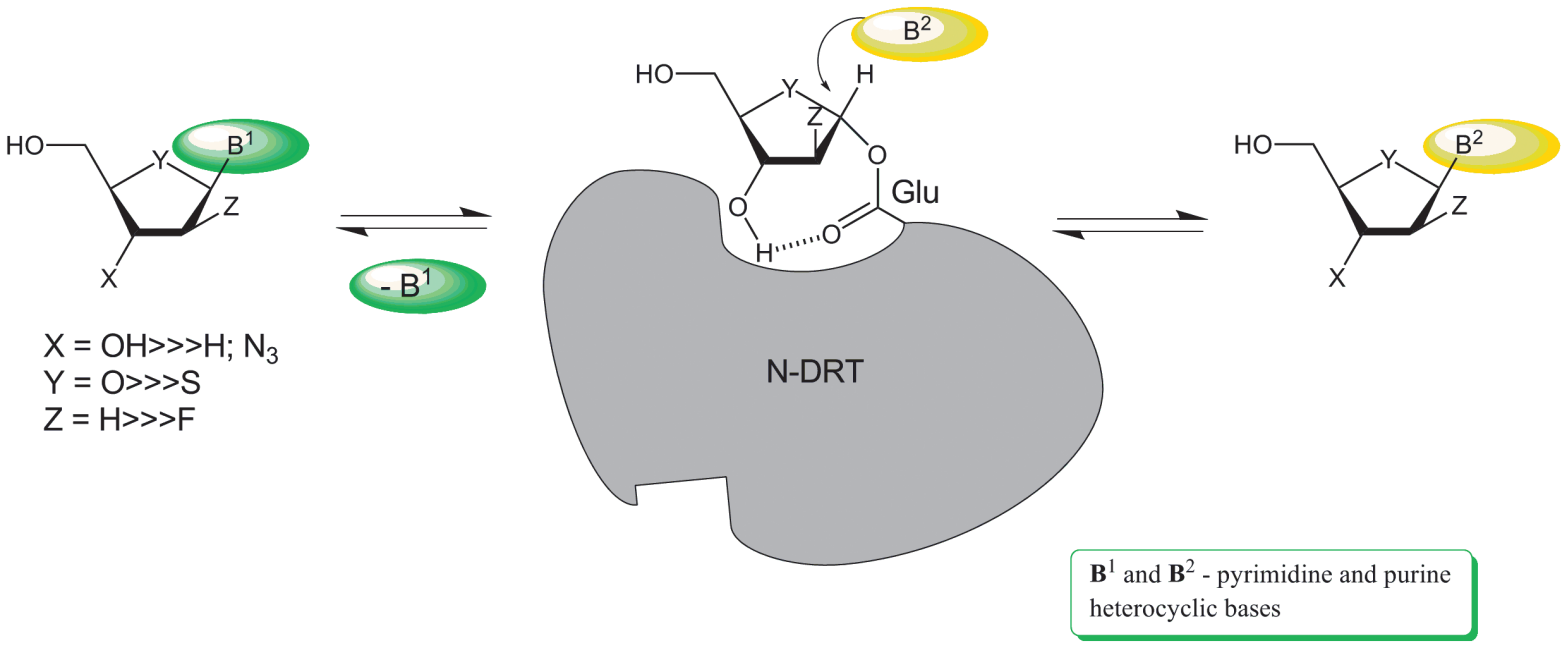


H. Shirae & K. Yokozeki isolated an orotidine–phosphorolysing enzyme (OrP) from * Erwinia carotovora * AJ 2992 and investigated its properties [91]. Orotidine was irreversibly phosphorolysed into orotic acid and 1–phosphate ** (47) ** by OrPE, and the enzyme showed no strict specificity. Indeed, the substrate activity of uridine was found to be two orders of magnitude higher as opposed to orotidine (relative activity of 100% and 1% for uridine and orotidine, respectively); moreover, 5–methyluridine (10%), aU (11%), 2 ′ –deoxyuridine (22%), 3 ′ –deoxyuridine (11%), and 2 ′ ,3 ′ –dideoxyuridine (1%) were also found to be substrates for the OrP preparation. At each purification step, OrPE was always co–purified with uridine phosphorylase (UP) and the researchers were unable to separate these two activities. Both activities corresponded to a single band on SDS–PAGE, suggesting that both activities are present in the same protein. The purified enzyme had a molecular weight of 68 000 ± 2 000 Da, which suggests a dimeric structure. The most interesting finding is that the optimal temperatures and the pH values of the phosphate buffer were found to be 60 °C and 6.0 for orotidine phosphorylase activity and 70 °C and 7.0 for the uridine phosphorylase activity. On the whole, despite the differences in the optimal conditions for these two activities, it appears that the enzyme preparation from *Erwinia carotovora * AJ 2992 consists of a UP with broad substrate specificity.

*N * –Deoxyribosyltransferases (DRT’s; nucleoside: purine(pyrimidine)deoxyribosyl transferases; EC 2.4.2.6) represent another type of enzymes, which are considerable interest as biocatalysts for nucleoside synthesis (for a review of pioneering studies, see [92]). As opposed to nucleoside phosphorylases, DRTs catalyze the direct transfer of the deoxyribofuranosyl moiety between a nucleoside and an acceptor base without intermediary formation of 2–deoxyribofuranose phosphate. The reaction proceeds through the intermediate formation of a covalently bound 2–deoxy– α – * D * –ribofuranosyl moiety, whose glycosidic hydroxyl forms a complex ester bond (Scheme 12) [93 and works cited in this paper].

DRTs are mainly present in some bacterial species of the * Lactobacillus* genus and were first discovered by W.S. MacNutt [94] in *Lactobacillus* *helveticus * and isolated by A. Roush & R. Betz [95]; later, DRT was purified from * L. leichmannii * by W. Beck & M. Levin, and its properties were thoroughly studied [96]. * Lactobacillus * bacteria contain DRT enzymes with two types of enzymatic activity, and these were first isolated by L. Holguin & R. Cardinaud from * L. helveticus * using affinity chromatography: DRT class I (also called purine deoxyribosyltransferase, PDT), which specifically transfer 2–deoxyribofuranose moieties from purine nucleosides to purine bases, and DRT class II (also called nucleoside deoxyribosyltransferase, NDT), which catalyze the transfer of 2–deoxyribofuranose between purines and pyrimidines in any combination [97]. Early reports on DRT substrate specificity revealed ( *i * ) strict specificity for the 2–deoxyribofuranose moiety, the absence of β – * D * –ribonucleoside substrate activity[92]; ( * ii * ) rather broad tolerance regarding various modifications of natural purines [96, 98, 99]; ( *iii * ) good substrate activity of cytosine as an acceptor of the 2–deoxy– and 2,3–dideoxyribofuranose residues, and the corresponding purine and pyrimidine nucleosides as donors of carbohydrate moieties [100] (for a review, see [24]).

A number of very interesting observations concerning the possible practical applications of DRT were made during the last two decades. Thus, D.A. Carson & D.B. Wasson investigated the substrate specificity of NDT isolated from * L. helveticus * (ATCC, #8018) (purified according to [96]) and found that the enzyme displays broad specificity both for pentofuranose residue donors and for purine and pyrimidine acceptors [100]. Testing the pentofuranose donor activity of 2 ′ ,3 ′ –dideoxy– β – * D* –nucleosides (ddN) in acetate buffer (pH 6.0) with an equimolar ratio between the donor and acceptor molecules at 37 °C revealed an exceptionally high activity of cytosine as an acceptor (16–60 nmol·min^–1^·mg^–1^ of enzyme with the following preference for donors: dT > ddG > ddC > ddA > ddI); donor activity of 2 ′ ,3 ′ –dideoxycytidine (ddC) and 3 ′ –deoxythymidine (dT) was found to be approximately 2.2–11.6 nmol·min^–1^·mg^–1^ of enzyme for adenine, guanine, and hypoxanthine acceptors.

**Scheme 13:**
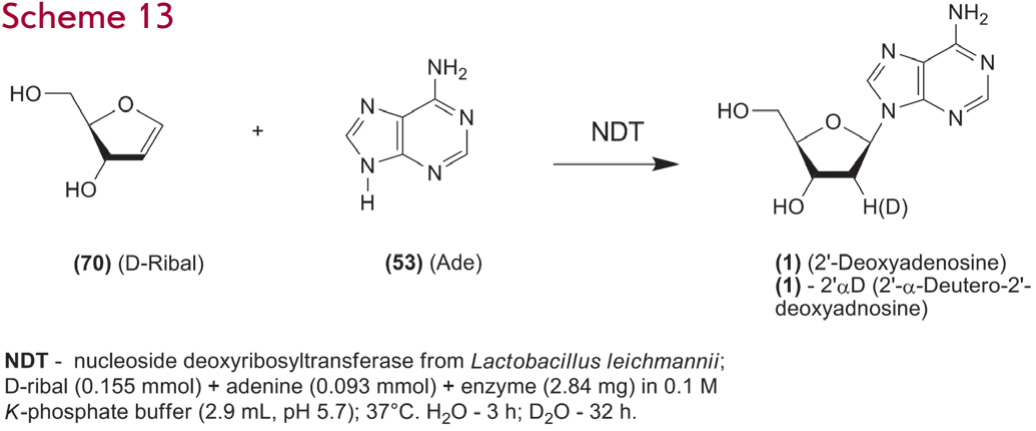


The first recombinant * L. leichmanii * NDT (DRT II) was prepared by W. Cook *et al * . [101]. These authors also studied the biochemical properties of this enzyme [102–104] and established the architecture of the enzyme’s active site [105]. In its native state, the enzyme turned out to be a hexamer composed of identical subunits with one active site per subunit, and two subunits forming a complete catalytic center. R. Wolfenden and co–workers discovered a lyase activity of * L. leichman* *ii* NDT and found that an interim 1– * O * –glutamyl derivative of 2–deoxy– * D * –ribofuranose is broken down in the absence of a heterocyclic base, yielding * D * –ribal. The latter reacts with adenine in a stereospecific manner under NDT catalysis, forming 2 ′ –deoxyadenosine in aqueous solution and its 2’– α –deuterium derivative in D_2_O (Scheme 13) [102]. Formation of thymidine and 2’–deoxyuridine from * D * –ribal and the respective bases could also be performed in a similar manner. The practical implications of this study of the chemoenzymatic synthesis of 2 ′ – β – *D * –deoxynucleosides have not yet been investigated; however, further studies in this direction seem practical, as * D * –ribal can readily be produced by chemical methods (see [106, 107]), and the recombinant enzyme is also available.

Notably, recombinant * L. leichmanii * NDT catalyzes the stero– and regioselective transfer of 3–azido–2,3–dideoxyribofuranose from AZT to various 2–amino–6–substituted purine bases (50 mM * Na* –citrate buffer, pH 6.0; 50 °C, 21–28 days) yielding the corresponding purine *N *^ 9 ^ – β – * D* –nucleosides with moderate yields. The same enzyme was also employed as a biocatalyst for the synthesis of purine 4 ′ –thionucleosides [104]. 2 ′ –Deoxy–4 ′ –thiouridine (used as an anomere mixture obtained via chemical glycosylation of uracil) was used as a carbohydrate moiety donor for the transglycosylation of a number of purine bases, with NDT as a catalyst (50 mM citrate buffer, pH 6.0; 50 °C, 5 days). Individual 9–(2 ′ –deoxy–4 ′ –thio– β – * D * –ribofuranosyl) purines were isolated with yields in the range of 5–48% after laborious treatment and chromatography of the reaction mixtures. It is worth noting that the use of thymidine and purine nucleoside phosphorylases as biocatalysts for the transglycosylation reaction yielded negative results.

**Scheme 14:**
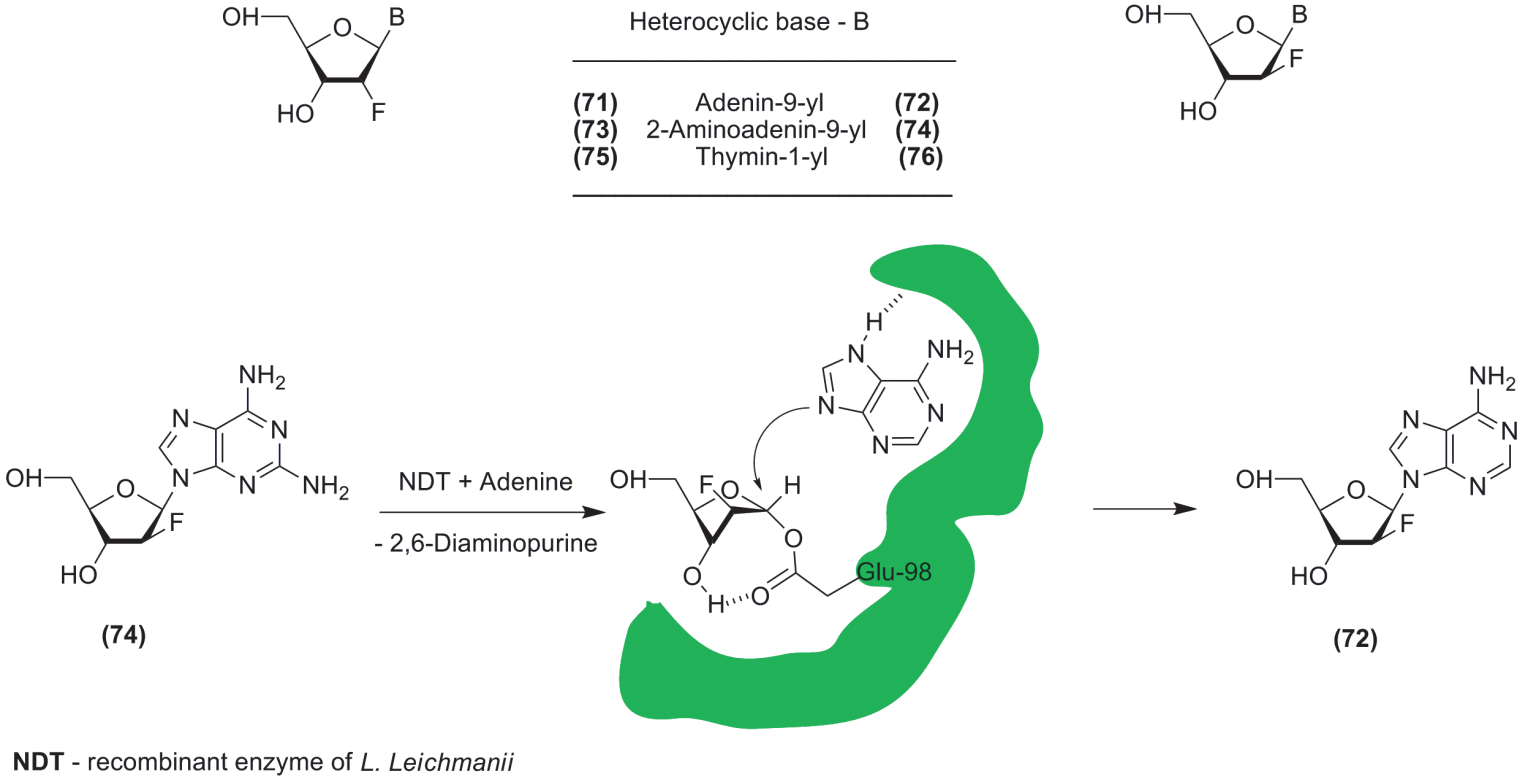


Identification of glutamic acid 98 as the active site nucleophile of recombinant * L. leichmanii * NDT (the DRT II class) was made by D. Porter * et al * . [108]. The authors thoroughly investigated the interaction of the enzyme with 4 isomeric pairs of nucleosides, namely 9–(2–deoxy–2–fluoro– β – * D* –ribo(arabino)furanosyl)adenine ( ** (71) ** and ** (72) ** ), 2–amino–9–(2–deoxy–2–fluoro– β – *D * –ribo(arabino)furanosyl)adenine ( ** (73) ** and ** (74)** ), 1–(2–deoxy–2–fluoro– β – * D* –ribo(arabino)furanosyl)thymine ( ** (75) ** and ** (76) ** ), and 9–( β – * D * – arabinofuranosyl)guanine ** (21;** aG ** ) ** . Incubation of the enzyme (2 microM) with arabinosyl nucleosides **(72) ** , ** (74), ** or aG ** (21) ** (100 microM) at 25 °C for 20 min resulted in inhibition of transferase activity by 91, 72, and 21%, respectively; thymine nucleosides did not inhibit the enzyme. The inhibited enzyme contained stoichiometric amounts of covalently bound 2–deoxy–2–fluoro– * D* –arabinose, and its activity could be restored upon treatment with adenine, which simultaneously yielded adenine arabinoside ** (72) ** . Proteolysis of the inhibited enzyme yielded data that suggest that the γ –carboxylate of Glu–98 is esterified during catalysis (Scheme 14). Finally, a recombinant enzyme, in which the Glu–98 residue is replaced by alanine, showed a decrease in activity by 3 orders of magnitude as compared to the wild–type recombinant enzyme.

Later on, P.A. Kaminski obtained recombinant * L. helveticus * PDT and NDT and determined that the polypeptides display 25.6% identity in the region involved in the binding of substrate to the Glu–98 residue of the enzyme’s active site [109]. Both enzymes catalyzed the transformation of 2–aminopurine and 2,6–diaminopurine into the corresponding 2–deoxy– β – * D * –ribonucleosides at a rate comparable to that of natural purine bases. 4–Aminoimidazole–5–carboxamide (AICA) and imidazole–5–carboxamide (ICA) turned out to be poor substrates, and their trans–2–deoxyribosylation required large quantities of enzyme and extended incubation times. It is worth noting that the specific activity of PDT was higher than that of NDT in all four studied transglycosylation reactions (no experimental details were given).

The structure of the recombinant purine 2 ′ –deoxyribosyltransferase of *L. helveticus * (PDT) was determined by X–ray crystallography [93], and the structure was found to be somewhat similar to that of NDT from * L. leichmanii * [105]. It was determined that, in the case of * L. helveticus* PDT, Glu–101 serves as the nucleophile in the active site, which attacks the glycoside carbon atom of the nucleoside, while the C3 ′ oxygen atom of the furanose moiety forms a hydrogen bond with one of the oxygen atoms in the carboxogroup of Glu–101 (Scheme 12). Glycosylated PDT, which is formed after treatment with adenine arabinoside ** (72),** contains a 2–deoxyfluoro– α – * D* –arabinofuranose residue covalently bound to one of the oxygen atoms of Glu–101. Comparison of the PDT–2’–deoxyadenosine and PDT–6–selenoinosine complex structures [105] allows to explain the specificity of the enzymes for 2 ′ –deoxynucleosides: namely that the C2’ and C3’ oxygen atoms of the ribonucleoside are involved in the formation of a hydrogen bond with Glu–101, making the formation of an intermediate structure with a covalently bound carbohydrate residue impossible (Scheme 12).

Recently, a very interesting study aimed at creating NDT with improved activity in regard to the synthesis of 2 ′ ,3 ′ –dideoxy purine nucleosides was published by Kaminski and co–workers [110]. The authors constructed random mutant libraries of * ndt * genes from * L. leichmanii* ( * Ll * ) and * L. fermentum * ( * Lf* ) with a variable frequency of nucleotide substitutions (between 1 and 10 per sequence), developed a functional screening method, and selected the mutants, which were suited for the synthesis of 2 ′ ,3 ′ –dideoxynucleosides. Sequencing of the corresponding genes revealed a single mutation (G3A transition), which caused a small aliphatic amino acid to be replaced by a residue with a hydroxyl group, Ala–15 was substituted for Thr ( *L. fermentum * ) or Gly–9 for Ser ( * L. leichmanii * ), respectively. This single amino acid substitution was sufficient to enhance the substrate activity towards dideoxynucleosides. The authors concluded that the 2,3–dideoxyribosyl transfer activity requires an additional hydroxyl group at the 9^th^ ( * Ll* ) or 15^th^ ( * Lf * ) position, so as to overcome the absence of such a group in the corresponding substrate. Both artificial enzymes also displayed significantly improved transferase activity in regard to 2 ′ ,3 ′ –didehydro–2 ′ ,3 ′ –dideoxy– β –D–ribofuranosyl nucleosides. It was shown (without experimental details) that the *Lf * –NDT A15T enzyme catalyzed the synthesis of 2 ′ ,3 ′ –didehydro–2 ′ ,3 ′ –dideoxyadenosine and 2 ′ ,3 ′ –didehydro–2 ′ ,3 ′ –dideoxyinosine using 2 ′ ,3 ′ –didehydro–2 ′ ,3 ′ –dideoxyuridine (d4U) as a donor of the pentofuranose moiety at the mM scale and with a good yield (up to 70%) [110].

Comparison of transglycosylation reactions catalyzed by a crude enzyme (NDT) preparation from *L. helveticus * [111] and * E. coli * purine nucleoside phosphorylase (PNP; Sigma) yielded rather unexpected results [112, 113]. On the whole, it was shown that NDT–catalyzed reactions proceeded with higher regioselectivity as compared to those catalyzed by PNP, and the difference strongly depended on the structure of the acceptor–base (for details, see [24]).

*N * –deoxyribosyltransferses are not restricted to *Lactobacilli * and have also been isolated from the protozoan parasites *Critinia lucilliae * (see, * e.g * ., [109]) and * Trypanosoma brucei brucei * [114, 115]. The enzyme from *T. b. brucei * was purified over 400–fold to >95% homogeneity from the bloodstream form of this parasite, and its properties have been investigated [79]; a recombinant enzyme of the same origin was also prepared [80]. As opposed to * Lactobacilli* enzymes, the enzyme from * T. b. brucei * was found to be * N* –ribohydrolase with a preference towards inosine, adenosine, and guanosine as substrates. The * k *_ cat _ / * K *_ m _ values for the recombinant enzyme and inosine, adenosine, and guanosine as substrates were ( × 10^6^ M^–1^·s^–1^) 1.6, 1.4, and 0.7, respectively. Pyrimidine and 2’–deoxynucleosides were poor substrates with * k *_ cat_ / * K *_ m _ values approximately 10^3^ M^–1^·s^–1^ and 10^2^ M^–1^·s^–1^, respectively. 3–Deazaadenosine, 7–deazaadenosine (Tubercidin), and formicin B were found to be inhibitors with *K_i_* values of 1.8, 59, and 13 microM respectively. To the best of our knowledge, this enzyme has not been used for the synthesis of nucleosides yet.

To sum up all of the above, we must note that chemo–enzymatic (biotechnological) strategies are currently displacing multi–stage chemical processes, and this allows key transformations to be achieved with high selectivity and regio– and stereospecificity. Considerable progress in the production of biologically important analogs of natural nucleosides has been achieved through the rational combination of chemical and biochemical transformations. Use of recombinant nucleoside phosphorylases and * N* –deoxyribosyl transferases as biocatalysts for the synthesis of natural nucleosides and their modified analogs is of considerable importance for the creation of modern technological processes. We must also note that the two enzymatic groups complement one another and allow finding out a straightforward way to the desired compound. The use of chemo–enzymatic methods undoubtedly allows improvement of the price–quality ratio during the production of many medical drugs.

## New Trends in Biotechnology of Nucleosides

A number of studies published over the last decade give new impulse for the development of nucleoside biotechnology. Much attention is given to the use of α – * D* –pentafuranose–1–phosphates as substrates for the enzymatic synthesis of nucleosides. It must be noted that both the enzymatic and chemical syntheses of *D * –pentofuranose–1–phosphates have extensive histories (see [24]). However, only several recently published works are interesting from a practical point of view. There are two main lines of research in this field: ( * i * ) biochemical (microbiological, enzymatic) * retro* –synthesis of 2 ′ –deoxyribonucleotides and ( * ii* ) chemical synthesis of * D * –pentofuranose–1–phosphates and their subsequent enzymatic condensation with heterocyclic bases.

**Scheme 15:**
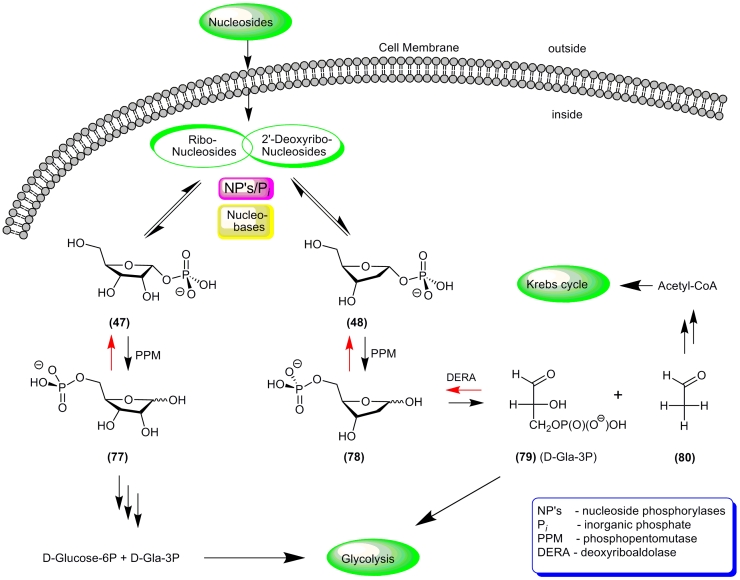


The metabolic transformations of pentoses have been investigated thoroughly (for a review, see, * e.g * ., [116]). Both in bacteria and eukaryotic cells nucleosides are regarded as carriers of carbohydrates, which serve as sources of carbon and energy. α – * D* –Ribofuranose–1–phosphate ** (47) ** is **** mainly produced from purine nucleosides via a process catalyzed by PNP. This phosphate is then involved in ( * i * ) glycolysis, ( * ii * ) metabolic activation of pyrimidine heterobases, which results in the formation of ribonucleosides ( * e.g* . transformation of 5–fluorouracil into 5–fluorouridine catalyzed by UP), and ( * iii * ) an enzymatic transformation into 5–phospho–D–ribofuranose, catalyzed by phosphopentomutase (PPM). This process is usually in a state of enzymatic equilibrium, and the product of this reaction (5–phospho–D–ribofuranose ) is a precursor of 5–phospho– α – * D * –ribofuranosyl–1–pyrophosphate (PRPP). The latter acts as a donor of the 5–phospho– * D * –ribofuranose moiety for both * de novo * and “salvage” synthesis of nucleosides. Catabolic transformations of 2 ′ –deoxynucleosides also proceed under the control of nucleoside phosphorylases and PPM, and the resulting 2–deoxy– * D* –ribofuranose–5–phosphate ** (78) ** is then irreversibly metabolized into * D * –glyceraldehyde–3–phosphate ( **(79) ** ; Gla–3P) and acetaldehyde ** (80) ** by bacterial or eukaryotic deoxyriboaldolases (Scheme 15).

**Scheme 16:**
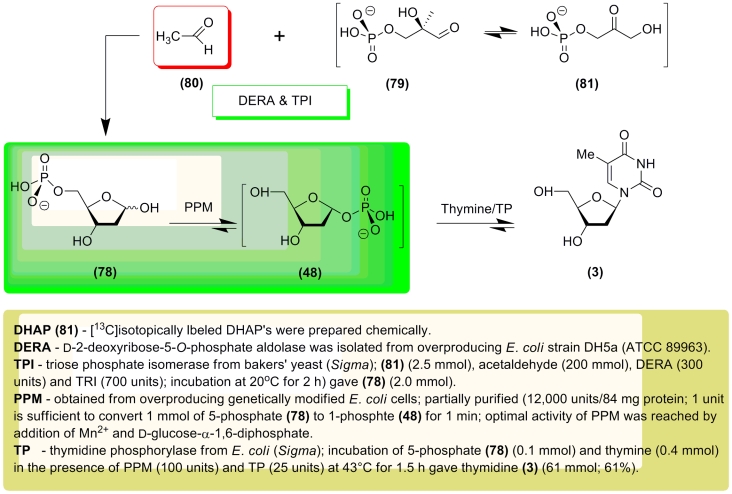


The reversed * retro * –pathway for nucleoside synthesis beginning with Gla–3P and acetaldehyde was studied by J. Raap and co–workers [117, 118]. The authors described a one–pot two–step enzymatic reaction involving glycosylation of thymine or uracil (labeled by ^13^C and ^15^N atoms) using 2–deoxy– α – * D* –ribofuranose–1–phosphate ( ** (48) ** ; **** also ^13^C–labeled at the different carbon atoms) and commercially available thymidine phosphorylase (TP). Synthesis of 1–phosphate ( ** 48 ** ) was performed using 2–deoxy– * D* –ribofuranose–5–phosphate ( ** 78 ** ) by stereospecific phosphate C5 → C1 translocation catalyzed by partially purified recombinant phosphopentomutase. The ^13^C–labeled 5–phosphates were enzymatically prepared from chemically synthesized dihydroxyacetone monophosphate ( ** 81 ** ) in the presence of an excess of acetaldehyde using deoxyriboaldolase (DERA) and commercially available triose phosphate isomerase (TRI; from baker’s yeast). The ** (78) ** → ** (48)** transformation and condensation with thymine or uracil were carried out in a one–pot system, and the respective 2 ′ –deoxyribonucleosides were then isolated in yields of 50–60% (Scheme 16). It should be stressed that the great excess of acetaldehyde is necessary to prevent the cleavage of Gla–3P and to direct the metabolic reaction in the reverse synthetic direction.

A similar approach was used by J. Ogawa * et al * . for the synthesis of 2 ′ –deoxynucleosides from acetaldehyde and dihydroxyacetone monophosphate through the intermediate formation of 5–phosphate ** (78) ** [119, 120]. The authors selected the *Klebsiella pneumoniae * B–4–4 strain for clones that could efficiently synthesize 5–phosphate ** (78) ** , which was then transformed into 1–phosphate ** (48) ** in the presence of transformed * E. coli* pTS17/BL21 cells, expressing * E. coli * PPM. The 1–phosphate (without any isolation procedures) was then condensed with adenine in the presence of commercially available PNP, which yielded a ~1:16 mixture of 2 ′ –deoxyadenosine ** (1) ** and 2 ′ –deoxyinosine ** (82) ** . Formation of the latter as the major product is due to the presence of adenosinedeaminase (ADA) in the * E. coli * pTS17/BL21 cells. This enzyme deaminates the initially formed 2 ′ –deoxyadenosine ( ** 1** ). Notably, the * K. pneumoniae * B–4–4 strain tolerated high concentrations of acetaldehyde, which directs the reversible DERA–catalyzed reaction in the direction of 5–phosphate synthesis ** (78) ** .

Later on, Ogawa and co–workers combined the alcoholic fermentation system of baker’s yeast and the DERA–expressing * E. coli * cells for the synthesis of 5–phosphate ( ** 78 ** ) [121 – 124]. The procedure for the synthesis of 2 ′ –deoxyribonucleosides consisted of four steps: ***1 *** – baker’s yeast synthesize fructose–1,6–diphosphate (FDP) via alcoholic fermentation; *** 2 *** – the DERA expressing * E. coli * 10B5/pTS8 cells transform FDP into an equilibrated mixture of dihydroxyacetone monophosphate ( ** 81 ** ) and * D* –glyceraldehyde–3–phosphate ( ** 79 ** ); enzymatic condensation of ( ** 79 ** ) and acetaldehyde (the high concentration of acetaldehyde is necessary in order to prevent the reversed reaction!) produces 5–phosphate; *** 3*** – the latter is transformed into 1–phosphate ( ** 48** ) under catalysis of PPM–expressing * E. coli * BL21/pTS17 cells; and finally step *** 4 *** , accomplished in one pot in the presence of a heterocyclic base and commercially available purine nucleoside phosphorylase or thymidine phosphorylase, since the activity of both enzymes within the used * E. coli* cells was insufficient.

Synthesis of 2’–deoxyadenosine ( ** 1 ** ) was also accompanied by the formation of 2’–deoxyinosine ( ** 82 ** ). Xylene and polyoxyethylenelaurilamine were used in order to improve the permeability of the * E. coli * cells, which in turn improved the yield of 5–phosphate (**78**).

Notably, microbial synthesis [119 – 124] appears to be limited to the production of 2 ′ –deoxy– β – * D * –ribonucleosides (isolation of individual products has not been published as of now). Also, satisfactory solubility of heterocyclic bases in the reaction mixture is an important prerequisite for successful nucleoside synthesis (for instance, low solubility of guanine makes synthesis of 2 ′ –deoxyguanosine highly improbable). It is also important to bear in mind the off–pathway activities present in the employed cells, which can prevent efficient synthesis of the desired product.

**Scheme 17:**
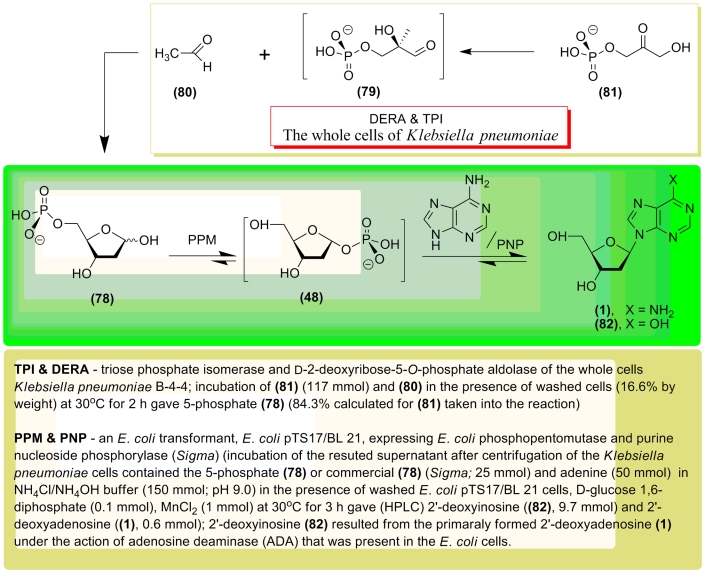


**Scheme 18:**
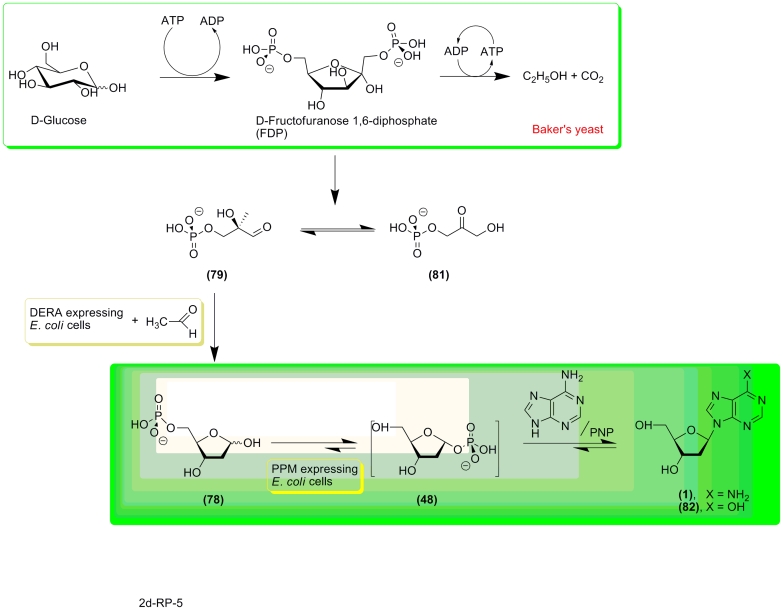


The second line of research, chemo–enzymatic synthesis, involves chemical synthesis of α – * D * –pentofuranose–1–phosphates, which are then used for enzymatic condensation with heterocyclic bases. This line of research presents more possibilities for variety and is promising for the synthesis of biologically important nucleosides and their analogs with modifications in the carbohydrate and base fragments. Indeed, α – * D * –pentofuranose–1–phosphates are universal glycosylation agents and can be used for the synthesis of both purine and pyrimidine nucleosides, as well as for reactions with any other type of heterocyclic base which can act as a substrate for nucleoside phophorylases.

**Scheme 19:**
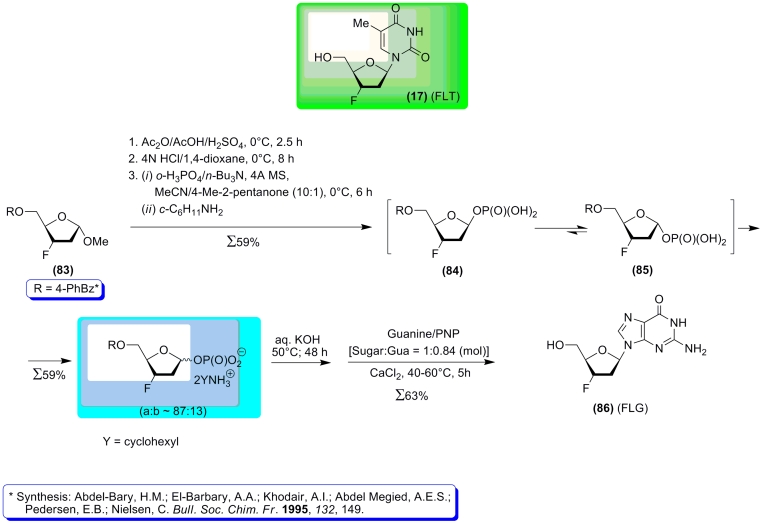


The effectiveness of this strategy was demonstrated in a very convincing manner almost simultaneously with the discovery of nucleoside phosphorylases and * N* –deoxyribosyl transferases. In this context, we must also note the pioneering studies on phosphorolysis and resynthesis of purine 2’–deoxyribosides involving mammalian nucleoside phosphorylases [40–49], purification of 2’–deoxy– α – * D * –ribofuranose–1–phosphate ( ** 48 ** ) as crystalline cyclohexylammonium salt [24, 53], and the synthesis of thymidine and a number of 5’–modified pyrimidine 2 ′ –deoxyribonucleotides [57, 58]. Further studies also managed to create procedures for the chemical synthesis of α – and β –anomers of *D * –ribofuranose–1–phosphate and 2–deoxy– *D * –ribofuranose–1–phosphate (see [24]).

A group of researchers from Mitsui Chemicals is also investigating the synthesis of nucleosides via condensation of α – * D* –pentofuranose–1–phosphates with heterobases using nucleoside phosphorylases [125–127]. First of all, they have developed “crystallization–induced asymmetric transformation” for the stereoselective synthesis of 2–deoxy– α – * D * –ribofuranose–1–phosphate ( ** 48** ) and its β – * D * –anomer [125, 126]. Both anomers have been isolated as pure stable bis(cyclohexylammonium) salts. It was also clearly shown that the former is a substrate for PNP, while the β – * D * –anomer did not show any substrate activity, as was expected. 2–Deoxy– α – *D * –ribofuranose–1–phosphate ( ** 48 ** ) was used for the synthesis of 2 ′ –deoxy–2–chloroadenosine (Cladribine) via one–step condensation with 2–chloroadenine or via a two–step process involving the intermediary formation of 9–(2–deoxy– β – * D* –ribofuranosyl)–2,6–dichloroadenine [128]. This method was then successfully extended to the synthesis of 2,3–dideoxy–3–fluoro–5– * О* –[(4–phenyl)benzoyl]– * D* –ribofuranose–1–phosphate (in the form of a ≈ 87 : 13 mixture of the α – and β –anomers ( ** 85) ** and ( ** 84 ** )) from methyl–2–deoxy– * D * –ribofuranoside ( ** 83** ), and the α –anomer from this mixture was then used as the main PNP substrate (after the removal of the 5– * О * –blocking group) for the synthesis of 2 ′ ,3 ′ –dideoxy–3 ′ –fluoroguanosine ( ** 86 ** ) via enzymatic glycosylation of guanine (Scheme 19) [127, 129].

This study is of vast importance for further development of this field of research, since it gives a clear answer to the following question: if the potential carbohydrate–modified nucleoside donor shows extremely low substrate activity towards the relevant nucleoside phosphorylase (like FLT ( ** 17 ** ) towards TP and UP) does this mean that the corresponding α – * D * –pentofuranose–1–phosphate (such as 2,3–dideoxy–3–fluoro– α – * D* –ribofuranose–1–phosphate) will also be lacking in substrate activity towards the same nucleoside phosphorylase? It is known that a number of pyrimidine nucleosides, which are easily synthesized via chemical methods, cannot act as substrates for TP and/or UP and thus cannot be used as pentose donors. Chemical synthesis of the appropriate α – *D * –pentofuranose–1–phosphates and assaying of their substrate qualities is of vast interest. The study by H. Komatsu * et al. * [127] is very revealing and demonstrates the need for further studies in this direction.

**Scheme 20:**
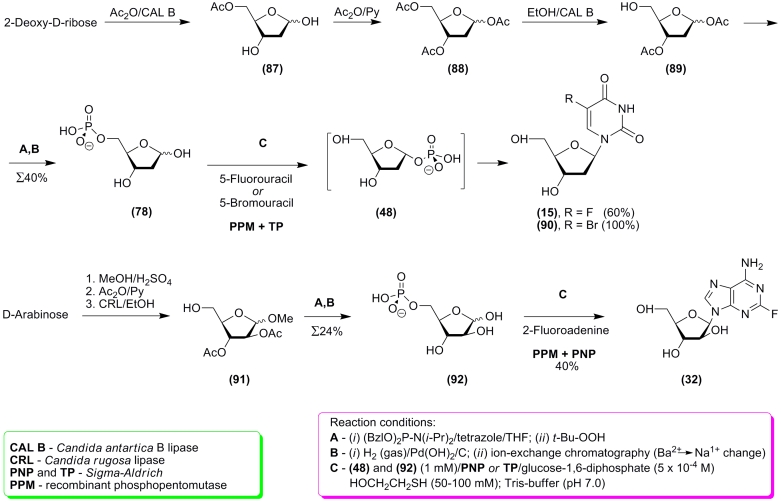


Recently, J.M. Montserrat * et al.* described a chemo–enzymatic approach to the nucleoside synthesis involving * D* –ribose, 2’–deoxy– * D * –ribose, and *D * –arabinose [130]. Pentoses were transformed into 5–phosphates (in the form of sodium salts) using chemical methods which sometimes utilized lypases for the introduction or removal of protective groups. The combined effect of PPM, which catalyzes the transformation of 5–phosphates into 1–phosphates, and condensation of the latter with heterobases in the presence of PNP or TP, leads to the formation of the appropriate nucleosides (Scheme 20).

The work of Montserra * t et al. * is very interesting as an example of rational chemo–enzymatic synthesis of * D* –pentofuranose–5–phosphates (compare the above work with [118, 119, 121–123]). We must of course note the universal approach to the synthesis of * D* –pentafuranose–5–phosphates, since the use of lypases for the regioselective introduction and removal of protective groups seems not to be limited to the studied pentoses.

**Scheme 21:**
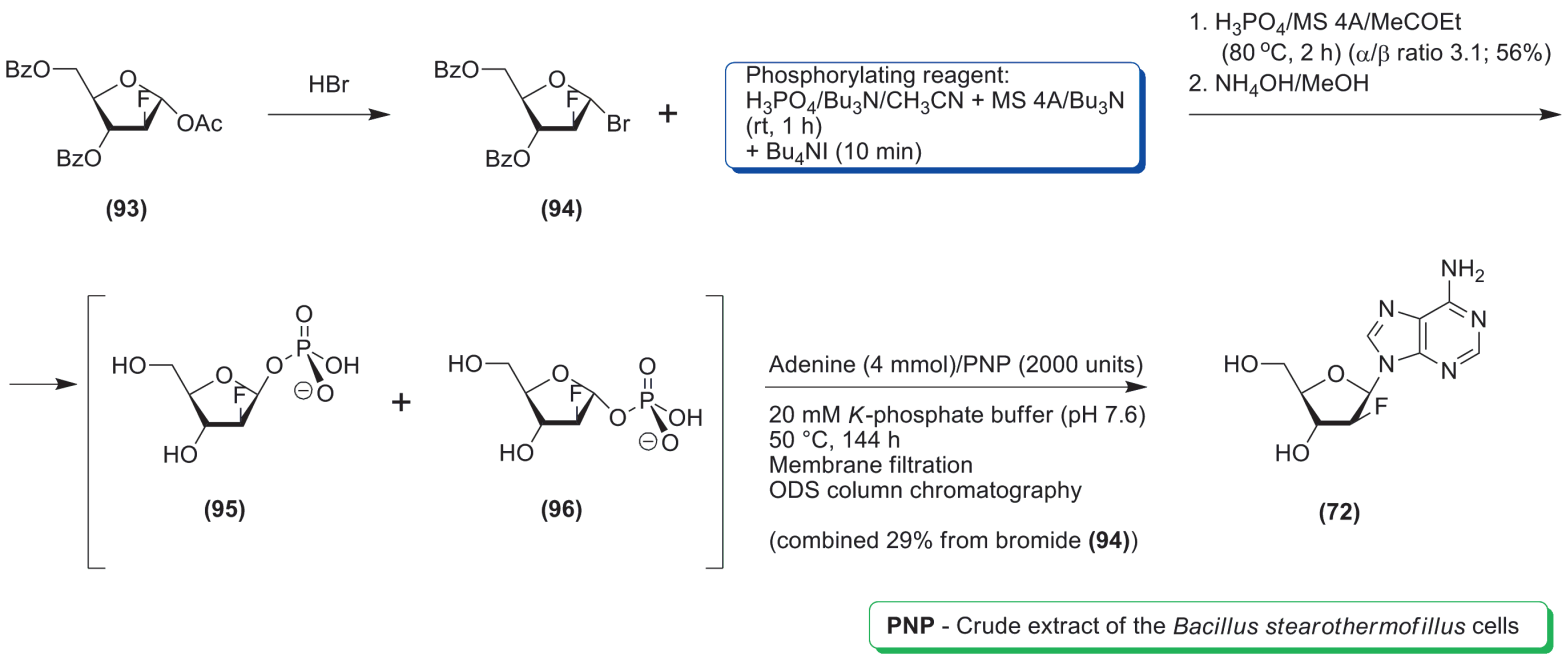


**Scheme 22:**
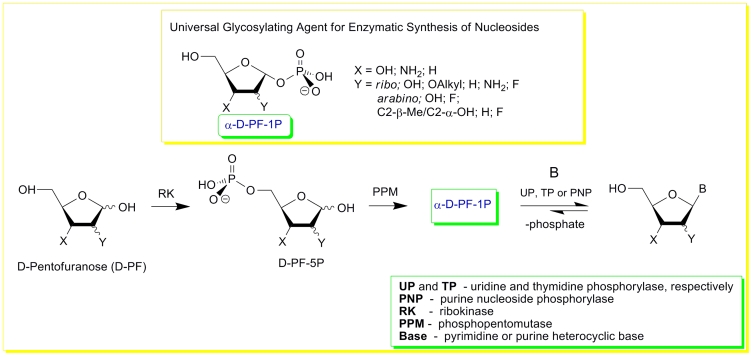


The synthesis of 9–(2–deoxy–2–fluoro– β – *D * –arabinofuranosyl) purines described in the study by K. Yamada *et al. * is also of considerable interest [131, 132]. In this case, there are no easy and simple methods for the synthesis of the potential carbohydrate fragment donor, which is why the chemical synthesis of 2–deoxy–2–fluoro– α – *D * –arabinofuranose–1–phosphate ( ** 96 ** ) and its use as a universal glycosylation agent seems to be a reasonable alternative to the chemical glycosylation of heterobases (Scheme 21). Commercially available 1– * О* –acetyl–3,5–di– * О* –benzoyl–2–deoxy–2–fluoro– α – * D* –arabinofuranose was used as the initial compound ( ** 93 ** ), which was then transformed into a bromide ( ** 94) ** and then into a ****** ≈ 3 : 1 mixture of α – and β –phosphates ( **96) ** and ( ** 95) ** . This mixture was used for the synthesis of *N^9^* –purine 2–deoxy–2–fluoro– β – *D * –arabinofuranosyl nucleosides without isolation of the individual α –anomer ( ** 96) ** , and the results were satisfactory. We must note that in some cases chemical glycosylation results in the formation of an anomeric mixture (purines and pyrimidines) and regioisomers (purines) [11, 12].

An analysis of the above–mentioned results leads to the conclusion that the laborious and low–yielding preparation of α – * D * –pentofuranosylphosphates is a serious bottleneck of this approach. However, despite this downside, it is an approach to the synthesis of biologically valuable nuclosides that is undoubtedly worthy of further investigation and is a valuable addition to the chemo–enzymatic methods reviewed above.

We recently proposed a novel nucleoside synthesis strategy which consists of the sequential transformation of pentoses into nucleosides in the presence of heterobases. The process is catalyzed by recombinant * E. coli * enzymes, namely ribokinase (RK) ( * D* –pentose → * D * –pentose–5–phosphate ( * D* –PF–5 * P * )), phosphopentomutase ( * D* –PF–5 * P * → α – * D* –pentofuranose–1–phosphate ( * D * –PF–1 *P * )), and nucleoside phosphorylases (NP) ( * D * –PF–1 *P * + heterobase → nucleoside) (Scheme 22) [133].

**Scheme 23:**
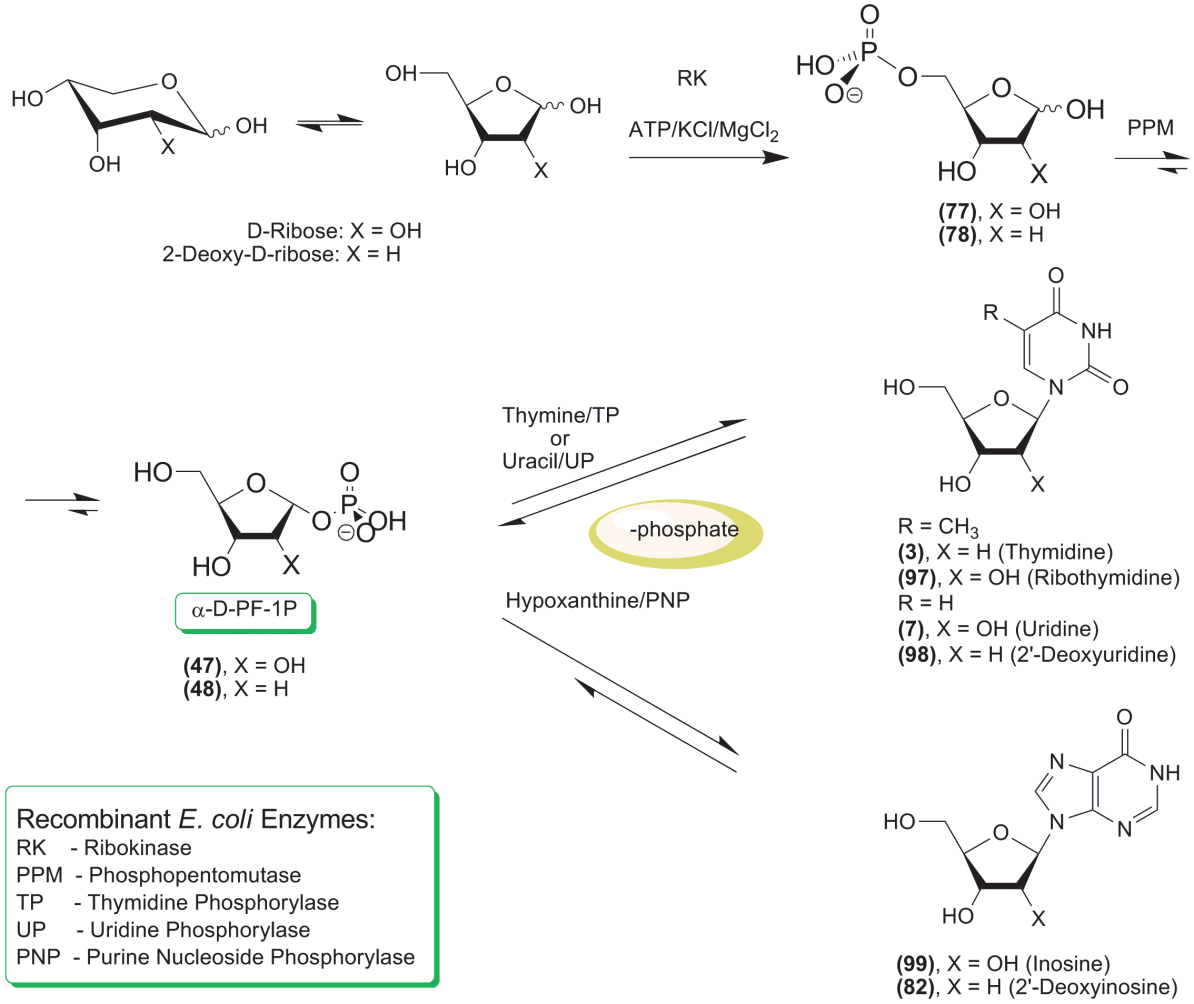


Production of recombinant RK, as well as that of uridine–, thymidine– and purine–nucleoside phosphorylases, was described in our previous work [134]. We observed that under optimal conditions RK can catalyze the phosphorylation of the primary hydroxyl group not only of *D * –ribose and 2–deoxy– * D * –ribose, but also of * D * –arabinose and * D * –xylose. These data suggest that RK may be used as a biocatalyst for the first step of the cascade transformation of pentoses into nucleosides. Stereospecific C5 → C1–translocation of phosphate by PPM is a reliable bridge within the proposed by us strategy of transformation of pentose into nucleoside, and this was the reason to produce recombinant PPM. The preliminary results of the transformation of * D * –ribose or 2–deoxy– *D * –ribose into pyrimidine and purine nucleosides using purified recombinant * E. c* *oli * RK, PPM, and nucleoside phosphorylases were recently published (Scheme 23) [135].

An analysis of the optimal reaction conditions for RK [133], PPM, and NP [134] showed considerable differences. Bearing this in mind, compromise conditions were chosen for a one–pot cascade transformation of pentoses into nucleosides. These conditions allow for the satisfactory activity of all the used enzymes and are as follows: overall volume of the reaction mix 2 ml; contents of the buffering solution: 2 mM ATP, 50 mM KCl, 3 mM MnCl2, 20 mM Tris–HCl (pH 7.5), 2 mM pentose, 2 mM heterobase; reaction temperature 20 °C; and enzymes (in the appropriate units): RK 7.65; PPM 3.9; TP 4.5; UP 5.4; PNP 4.68. The results of the * D * –ribose and 2–deoxy– *D * –ribose transformation into pyrimidine and purine nucleosides are presented in Scheme 23 and Table 1.

**Scheme 24:**
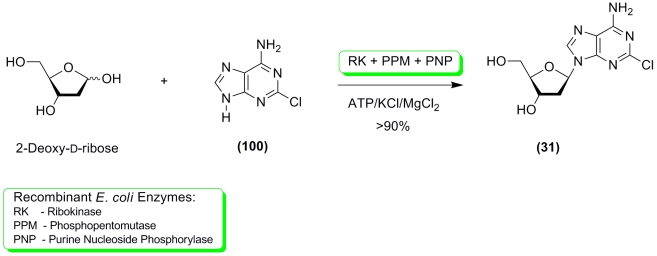


Notably, inosine is formed at a faster rate compared to 2 ′ –deoxyinsoine, and the maximum yield is achieved after 30 minutes. Also, the synthesis of purine deoxyribonucleotides was much more effective under transglycosilation conditions as compared to ribonucleside synthesis [82–84]. Obviously, the studied conditions for the cascade transformation of pentoses into nucleosides require thorough optimization for higher yields of the desired products. Showcase synthesis of Cladribine ( **31 ** ) shows that a 1.5 : 1 mixture of 2–deoxy– * D* –ribose and 2–chloroadenine ( ** 100 ** ) (mole/mole) substrates results in a product yield in excess of 90% (Scheme 24) [136].

**Table 1:** Progress of nucleoside syntheses in cascade one-pot enzymatic reactions at 20°C [content of the corresponding nucleoside (%) in the reaction mixture vs time of reaction].

Time of Reaction, h	Inosine (Ino)	2’-Deoxy-inosine (dI)	Thymidine (Thd)/2’-Deoxyuridine (dU)^[Table-fn TF1-1]^	1-(β-D-Ribofuranosyl)thymine (Rib-Thy)/Uridine(Urd)^[Table-fn TF1-1]^
0.5	45.9	18.8	14.5/0.9	4.7/27.6
1	46.1	27.3	17.6/1.1	8.5/26.6
24	38.4	38.3	-	-
44	-	-	34.7/33.2	19.9/17.5
96	29.4	34.4	-	-

Thymidine (TP) and uridine (UP) phosphorylases were employed for the synthesis of thymine and uracil nucleosides, respectively.

As was noted earlier, chemical synthesis of α – * D* –pentofurnanose–1–phosphates is relatively complex, which means that these compounds will probably not gain wide–spread in for the production of preparative amounts of nucleosides. Preliminary results of the cascade transformation of pentoses into nucleosides using three enzymes indicate that this strategy is worth investigating further in terms of its limitations and possibilities for use.

A survey of the chemical methods for the production of pento(hexo)–furanose–1–phosphates [125–132, 137–147] and the methods of anomeric carbon atom activation (see [139] for a review) shows that most of these methods are laborious and low–yielding of the desired phosphates. As can be expected, most of the procedures yield mixtures of anomers, and it seems that only the “crystallization–induced asymmetrical transformation” preferably yields the desired 2–deoxy– α – * D* –pentofuranose–1–phosphates [85].

**Scheme 25:**
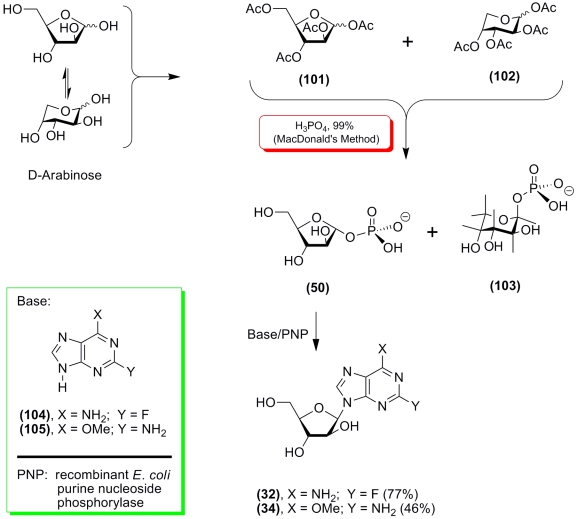


Because of its relative simplicity, the method proposed by * D.L. MacDonald* [140–144] seems to be the most effective, which is why we chose to use it for the synthesis of α – * D * –pentofuranose–1–phosphates. The method proposed by MacDonald is effective for the synthesis of hexopyranose–1–phosphates and was also used for the synthesis of α – *L * –arabinofuranose–1–phosphate in a study by G.O. Aspinall *et al * .: incubation of a peracetyl–derivative of * L* –arabinofuranose (mixture of α –, β –anomers) in anhydrous phosphoric acid and anhydrous THF at 50°C for 2 h yielded a mixture of * L* –arabinofuranose–1–phosphate (mostly α – * L* –anomer) and * L * –arabinopyranose–1–phosphate (both in the form of cyclohexylammonium salts) in an overall yield of 19% [147]. However, there were no conclusive physico–chemical data in support of the indicated structures.

Bearing in mind that a large number of purine and pyrimidine β – * D * –arabinofuranosides exhibit strong antiviral and antitumor activity (see above and also [18, 19, 148–150]), we chose the MacDonald approach for the synthesis of * D* –arabinofuranose–1–phosphate and used it for the synthesis of purine nucleosides.

A freshly prepared * D * –arabinose tetraacetate was a mixture of α –, β –anomers of furanose ( ** 101 ** ) and pyranose ( ** 102 ** ) forms (compare with [151]); treatment of this mixture according to the MacDonald method yielded an amorphous mixture of α – * D–* arabinofuranose–1–phosphate ( ** 50 ** ) and β – * D* –arabinopyranose–1–phosphate ( ** 103 ** ) (overall yield ≈ 50%; isomer ratio from 1.5 : 8 to 1 : 2, as assayed by ^1^H–NMR). This mixture was tested in reactions with 2–fluoroadenine ( ** 104 ** ) and 2–amino–6–methoxypurine ( ** 105 ** ), catalyzed by recombinant *E. coli * PNP.

We observed that pyranose 1–phosphate ( **102 ** ) does not inhibit the synthesis of 9–( β – * D* –arabinofuranosyl)–2–fluoroadenine (( ** 32) ** ; Fludarabine) under optimal conditions (water solution, pH 7.0, 55 °C; 1 hour). This procedure had a yield of 77% (Scheme 25) [152]. Unexpectedly, the rate of Fludarabine formation was similar to the rate of 2–fluoroadenosine synthesis from α – * D * –ribofurnaose–1–phosphate (Sigma) and 2–fluoroadenine ( ** 104 ** ) in the presence of recombinant PNP extracted from *E. coli * .

The high rate of Fludarabine formation was unexpected (compare with [130]). In chemical terms, the condensation of α – * D–* pentofuranose–1–phosphates with heterobases is the result of a nucleophilic attack of the heterobase nitrogen atom on the electrophilic anomeric carbon atom of the 1–phosphate. In order to asses the electrophilic properties of the С 1–atom, we used an * ab initio * method for the geometry optimization of a number of related phosphate structures, namely α – * D* –ribofuranose–1–phosphates (( ** 47) ** ; Rib * f* – α 1 * P * ), α – * D* –2–deoxyribofuranose (( ** 48) ** ; dRib * f * – α 1 * P * ); and (( ** 50) ** Ara * f * – α 1 * P * ) (Table 2).

It follows from the data in Table 2 that the positive partial charges of the С 1–atoms of 2– *deoxyribo * – and * arabino * –phosphates are similar in value and are stronger than the charges of the * ribo* –isomer. The latter has the C–2 hydroxyl and phosphate group in * cis–* conformation and is more stable than the * arabino * –phosphate. The spatial structures of the * ribo– * and 2– * deoxyribo* –phosphates are more favorable for nucleophilic attack, and the С 2–hydroxyl of the * arabino * –isomer does not create significant steric barriers for the approach of the base towards the С 1–atom [152].

Differences in the partial positive charge of the С 1–atoms of * ribo * – and * 2–deoxyribo* –phosphates are confirmed by the fact that trans– * deoxyribo* sylation is more effective than trans– * ribo * sylation of deazapurines [24, 82] and benzimidazoles [24, 83, 84]. A similar substrate activity of Rib * f * – α 1 * P * and Ara *f * – α 1 * P * in a reaction with 2–fluoroadenine can seemingly be explained by two interacting factors: the high partial positive charge of the С 1–atom of Ara * f * – α 1 *P * , on the one hand, and the negative steric effect of the С 2–hydroxyl, on the other (compare this with data from [130]).

It shoud be noted that calculations indicate that both conformers of β – * D* –arabinopyranose–1–phosphate, namely 4 * C^1^* and ^4^ *C_1_* , have higher thermodynamic stability as compared to Ara * f * – α 1 * P * . These differences seem to account for the preferential formation of pyranose phosphate during the MacDonald reaction.

Unlike 2–fluoroadenine, a reaction between 2–amino–6–methoxypurine ( ** 105 ** ) and Ara * f* – α 1 * P * (in a mixture with Ara * p * – β 1 * P * ) in the presence of recombinant * E. coli * PNP under conditions specified earlier reached equilibrium at an equimolar ratio between the initial heterobase and reaction product, 2–amino–9–( β – *D * –arabinofuranosyl)–6–methoxypurine (( ** 34) ** ; Nelarabine), which could then be isolated in a yield of 44%. This result is in accordance with an earlier Nelarabine synthesis in a yield of 53% and involved the transarabinosylation of 2–amino–6–methoxypurine ( ** 105 ** ), using 1–( β – * D * –arabinofuranosyl)uracil ( ** 49 ** ) as a carbohydrate group donor and * E. coli * UP and PNP as biocatalysts [153].

We have observed earlier that trans–2–deoxyribosylation of * N *^ 2^ –acetylguanine with thymidine or 2 ′ –deoxyguanosine as a carbohydrate group donor and TP/PNP or PNP as a biocatalyst initially leads to the formation of *N^2^* –acetyl–7–(2–deoxy– β – *D * –ribofuranosyl)guanine, which eventually rearranges into the more thermodynamically stable * N^2^* –acetyl–9–(2–deoxy– β – * D* –ribofuranosyl)guanine [76]. On the contrary, the Fludarabine and Nelarabine syntheses did not involve similar reaction stages [152]. This result allows us to hypothesize that the electron structure of the heterocyclic base determines the heterobase’s mode of binding in the PNP active site, thus determining the regioselectivity of the enzymatic reaction.

**Table 2:** Results of the *ab initio* geometry optimization procedure (HyperChem, 8.1; *in vacuo*, 6-31G* level) for the spatial structures of α(β)-D-pentofuranose(pyranose)-1-phosphates (in mono sodium salt form).

Compound	Positive partial charge at the C1 carbon atom	Total (binding) energy kcal/mol	Conformation of the pento-furanose (pyranose) ring
[47]; Rib*f*-α1P	0.425	-808 850.3	C1-exo
[48]; dRib*f*-α1P	0.454	-762 140.7	C3-endo
[50]; Ara*f*-α1P	0.464	-808 841.6	O4-exo
[103]; Ara*p*-β1P	0.410	-808 868.5	_4_C^1^ (more stable)
	0.451	-808 856.8	_4_C^1^ (less stable)

## CONCLUSIONS

An analysis of the results of the chemoenzymatic syntheses of nucleosides clearly indicates that this methodology is highly effective and very promising for the development of biotechnological processes for the production of biologically important compounds. Glycosylation of heterocyclic bases is catalyzed by two types of enzymes: nucleoside phosphorylases and * N * –deoxyribosyl transferases. These enzymes exhibit varying substrate specificities, which is why they mutually complement in terms of their use as biocatalysts.

Overall, all the above–mentioned results demonstrate the clear advantages of enzymatic methods for nucleoside synthesis as opposed to chemical methods. First of all, enzymatic methods fully conform to the principles of “green chemistry,” since routinely they do not use aggressive reagents (apart from acetic aldehyde) or organic solvents. Secondly, the high effectiveness of enzymatic transformations and their stereo– (only β – * D * –nucleosides!) and regioselectivity (apart from some specific cases) simplify the production of the desired compounds and increase the product’s quality. All of these factors lower the costs of production of biologically important compounds, making these compounds more available for researchers, and making drugs more available for widespread use.
